# Exploring individual differences in infants’ looking preferences for impossible events: The Early Multidimensional Curiosity Scale

**DOI:** 10.3389/fpsyg.2022.1015649

**Published:** 2023-02-02

**Authors:** Nayen Lee, Vanessa Lazaro, Jinjing Jenny Wang, Hilal H. Şen, Kelsey Lucca

**Affiliations:** ^1^Department of Psychology, Arizona State University, Tempe, AZ, United States; ^2^Department of Psychology, University of Chicago, Chicago, IL, United States; ^3^Department of Psychology, Rutgers University, New Brunswick, NJ, United States; ^4^Faculty of Psychology, University of Akureyri, Akureyri, Iceland; ^5^Department of Psychology, MEF University, Istanbul, Turkey

**Keywords:** curiosity, exploration, scale development, parenting, awe, cognitive development, individual differences

## Abstract

Infants are drawn to events that violate their expectations about the world: they look longer at physically impossible events, such as when a car passes through a wall. Here, we examined whether individual differences in infants’ visual preferences for physically impossible events reflect an early form of curiosity, and asked whether caregivers’ behaviors, parenting styles, and everyday routines relate to these differences. In Study 1, we presented infants (*N* = 47, *M_age_* = 16.83 months, *range* = 10.29–24.59 months) with events that violated physical principles and closely matched possible events. We measured infants’ everyday curiosity and related experiences (i.e., caregiver curiosity-promoting activities) through a newly developed curiosity scale, *The Early Multidimensional Curiosity Scale* (EMCS). Infants’ looking preferences for physically impossible events were positively associated with their score on the EMCS, but not their temperament, vocabulary, or caregiver trait curiosity. In Study 2A, we set out to better understand the relation between the EMCS and infants’ looking preferences for physically impossible events by assessing the underlying structure of the EMCS with a larger sample of children (*N* = 211, *M_age_* = 47.63 months, *range* = 10.29–78.97 months). An exploratory factor analysis revealed that children’s curiosity was comprised four factors: Social Curiosity, Broad Exploration, Persistence, and Information-Seeking. Relatedly, caregiver curiosity-promoting activities were composed of five factors: Flexible Problem-Solving, Cognitive Stimulation, Diverse Daily Activities, Child-Directed Play, and Awe-Inducing Activities. In Study 2B (*N* = 42 infants from Study 1), we examined which aspects of infant curiosity and caregiver behavior predicted infants’ looking preferences using the factor structures of the EMCS. Findings revealed that infants’ looking preferences were uniquely related to infants’ Broad Exploration and caregivers’ Awe-Inducing Activities (e.g., nature walks with infants, museum outings). These exploratory findings indicate that infants’ visual preferences for physically impossible events may reflect an early form of curiosity, which is related to the curiosity-stimulating environments provided by caregivers. Moreover, this work offers a new comprehensive tool, the *Early Multidimensional Curiosity Scale,* that can be used to measure both curiosity and factors related to its development, starting in infancy and extending into childhood.

## 1. Introduction

Young children are exceptionally curious. They want to know how and why things work the way they do. This drive to seek out information propels early learning: by the time children enter school, they have an impressive understanding of how the world around them works. And critically, when children encounter information that contradicts what they already know, they actively work to update their knowledge, either by asking questions or directly exploring their environment (Jirout and Klahr, 2012; Ronfard et al., 2018; Lucca, 2020). What makes children this way? Where does this seemingly endless drive for new information come from?

Though it has long been known that very young infants are drawn to informationally rich stimuli (e.g., novel toys, human faces, and infant-directed speech; Hunter et al., 1983; Cooper and Aslin, 1990; Morton and Johnson, 1991), research beyond infants’ basic preferences was scarce. Historically, it was widely presumed that infants did not have the prerequisite capacities (e.g., metacognition or information-seeking abilities) required for epistemic curiosity, that is, curiosity motivated by the drive to acquire new knowledge (Berlyne, 1960). Information theoretical approaches to curiosity have pointed to uncertainty as opportunities for information gain and therefore drivers of intrinsic curiosity (Gottlieb et al., 2013; Kidd and Hayden, 2015; Kobayashi et al., 2019). Uncertainty in children’s current mental states can cause discomfort and encourage them to explore and gather information that may potentially resolve the uncertainty (Lowry and Johnson, 1981; Bonawitz et al., 2011; Pluck and Johnson, 2011; Wang et al., 2021).

Recent research has made it increasingly clear that these forms of curiosity centered around resolving uncertainty that we see later in life are rooted in infancy (Harris et al., 2017; Lucca, 2020). Preverbal infants are more likely to spontaneously attend to stimuli with higher levels of uncertainty, either in the form of novelty (e.g., Weizmann et al., 1971), complexity (e.g., Kidd et al., 2012), or surprise (e.g., Stahl and Feigenson, 2015). Infants also seem sensitive to gaps in their knowledge—and when they reach such gaps (e.g., when their expectations are violated), they actively work to fill those gaps, either by exploring their environment or seeking out information from a knowledgeable social partner (Kovács et al., 2014; Goupil et al., 2016; Goupil and Kouider, 2016; Lucca and Wilbourn, 2018, 2019; Bazhydai et al., 2020). These findings point to an early origin of epistemic curiosity, but we still know very little about the basic nature of early curiosity and how it develops. Though early curiosity manifests itself in myriad ways (Kidd and Hayden, 2015), here we focus on infants’ motivation to fill knowledge gaps triggered by witnessing physically impossible events (Baillargeon, 1987; Spelke et al., 1992; Stahl and Kibbe, 2022).

### 1.1. Infants’ visual preference for physically impossible events: An early form of epistemic curiosity?

Long before they begin to speak, infants demonstrate a basic understanding of key principles that define the physical world, such as continuity (objects cannot blink in and out of existence; Aguiar and Baillargeon, 1999), support (objects cannot float without physical support; Baillargeon et al., 1992), and solidity (solid objects cannot pass through each other; Spelke et al., 1992). When infants view events that violate these expectations (e.g., a car passing through a solid wall), they show heightened visual attention compared to when they view events that are consistent with these expectations (e.g., a car stopping upon hitting a solid wall).

Crucially, infants appear to treat these expectancy violations as signals for potential information gain. Infants both spend more time exploring objects that they have seen previously violate physical principles, and show enhanced memory for these “impossible” objects compared to regular objects that did not violate any physical principles (Stahl and Feigenson, 2015). These findings suggest that infants’ looking preference for physically impossible events may reflect their drive to gather more information and fill their knowledge gaps.

Though on average infants look longer at physically impossible events relative to possible events, individuals vary greatly in their looking behavior (Wang et al., 2004; Luo and Baillargeon, 2005). A recent, ground-breaking study by Perez and Feigenson (2021) revealed that infants’ interest in impossible events is relatively stable over the first 2 years of life: the degree to which infants visually attend to solidity violations at 11 months of age predicts visual attention to support violations at 17 months. Critically, Perez and Feigenson (2021) also revealed that infants’ looking preference for physically impossible events at 17 months predicted parents’ ratings of children’s curiosity at age three.

Despite the importance of Perez and Feigenson (2021) study in laying the groundwork for individual differences in early epistemic curiosity, key questions remain. First, infants’ everyday curiosity was reported by parents only during early childhood (i.e., at 3 years old), not infancy, making it unclear whether individual differences in looking preferences during infancy already reflect concurrent everyday curious behaviors during infancy, or whether other factors (e.g., infant temperament) co-vary with infants’ looking preferences during infancy and explain the link to later curiosity. To understand the concurrent link between infants’ looking preferences and curiosity during infancy, there is a need to develop new methods for capturing everyday curiosity during infancy.

Second, Perez and Feigenson (2021) did not control, at the *individual level*, for infants’ preferences for physically possible events when examining links between preferences for physically impossible events and later curiosity. That is, they did not measure how long each infant attended to impossible *relative to* possible events. This raises the possibility that infants’ heightened visual attention to physically impossible events reflects general information-processing abilities instead of selective visual exploration targeted toward closing knowledge gaps.

A final open question that emerged from Perez and Feigenson (2021) experiment concerns predictors of individual differences in infants’ looking preferences for physically impossible events. What role, if any, does the home environment play in the development of these preferences? Answering these questions is critical for understanding whether and how infants’ responses to physically impossible events relate to individual differences in curiosity across development.

In the current study, we addressed these gaps in two ways. First, we developed a new, developmentally appropriate scale designed to measure infants’ everyday curious behaviors and related experiences (i.e., caregiver curiosity-promoting behaviors). Second, using this new scale, we explored whether infants’ everyday curiosity and related experiences provided by caregivers are associated with infants’ looking preferences for physically impossible events after controlling for possible confounding variables, such as temperament and general visual attention that were not included in Perez and Feigenson (2021) study.

### 1.2. New measures to capture infants’ everyday curiosity and caregiver curiosity-promoting behaviors

Though a number of curiosity scales have been validated with adults and older children (e.g., Piotrowski et al., 2014; Kashdan et al., 2018), a comprehensive curiosity scale for infants and toddlers has yet to be developed. Existing measures of infant curiosity are typically taken from a single assessment (e.g., exploratory play with a novel object), which is not only conflated with other constructs (e.g., temperament, problem-solving skills) but also too narrow to capture diverse expressions of curiosity (e.g., curiosity about people, information-seeking). Relatedly, there is a need for more systematic measures to comprehensively assess caregivers’ curiosity-promoting behaviors. Typically, caregiver curiosity-promoting behaviors are extracted from a short experimental setting (e.g., brief parent–child interactions during free play, Endsley et al., 1979; Medina and Sobel, 2020), which fail to capture a wide range of possible curiosity-promoting behaviors (e.g., activities caregivers routinely involve their children, such as exposure to new people and places).

In the current study, we fill these gaps by developing a new comprehensive scale, “The Early Multidimensional Curiosity Scale” (EMCS), that measures a range of both infant and caregiver behaviors, preferences, and traits that relate to early curiosity. Together, this is the first scale that takes a holistic approach to capture children’s early curiosity and related experiences. Rather than scoring infants and caregivers as “high” versus “low” on curiosity, this scale aims to measure multiple, diverse facets of curiosity, or “ways of being curious,” as is commonly done with adults (Kashdan et al., 2018; Wagstaff et al., 2021), and “ways of promoting curiosity,” which has yet to be explored in early childhood.

### 1.3. The multidimensionality of curiosity

Starting in childhood and extending across the lifespan, curiosity has been shown to be a multidimensional trait that draws on distinct underlying “curiosity factors” (Kreitler et al., 1975; Henderson and Moore, 1979; Kashdan et al., 2018). Each of these factors reflects distinct psychological processes and includes Joyous Exploration (the joy of discovering new information), Deprivation Sensitivity (frustration in the face of uncertainty), Stress Tolerance (ability to handle stress associated with confronting something new), Social Curiosity (interest in learning about others), and Thrill Seeking (willingness to take risks). These curiosity factors function differently within individuals, and predict unique aspects of well-being and everyday behavior. For example, joyous exploration, but not other dimensions of curiosity, predicts subjective happiness (Kashdan et al., 2018).

Though these factors may not have direct correlates with infant curiosity, we drew inspiration from these dimensions when designing the EMCS. Questions about infant curiosity in the EMCS asked about infants’ information-seeking behaviors (e.g., “When playing with a new toy, how often does your child communicate with you as if requesting help or further instruction?”), social preferences (e.g., “To what extent does your child enjoy interacting with new adults?”), reaction to novelty (e.g., “How likely is your child to prefer a familiar toy over a new toy?”), and broad exploration behaviors (e.g., “How likely is your child to explore the majority of toys in a room?”). Items in the survey also asked about psychological processes that are closely involved in curiosity, such as persistence (e.g., “How often does your child further explore, as if to figure out what happened when something contradicts what your child knows?”) and creativity (e.g., “How often does your child independently discover creative uses for objects outside of their intended purpose?”; Litman and Mussel, 2013; Evans et al., 2021).

Since there are multiple ways of being curious, there may also be distinct ways of promoting curiosity. Thus, to generate items measuring caregiver behavior for the EMCS, we asked parents about a range of different types of curiosity-related behaviors and activities. We selected behaviors shown to predict curiosity in older children, such as caregivers’ question-asking strategies (e.g., “How often do you ask your child open-ended questions?;” Medina and Sobel, 2020), encouragement of autonomy in exploration (e.g., “How often do you lead play time?”; Medina and Sobel, 2020), and exposure to novelty and uncertainty (e.g., “How often do you take your child to new places?”; Gottfried et al., 2016). We also asked about children’s exposure to awe-inducing experiences, as recent experimental work has linked the experience of awe to epistemic curiosity (Anderson et al., 2020; Paulson et al., 2021), and has even shown a causal link between exposure to awe and curiosity (Colantonio and Bonawitz, 2018). We selected awe-inducing experiences that past work has linked to curiosity, such as going to science or art museums (Gottfried et al., 2016) and spending time in nature (Keltner and Haidt, 2003; Valdesolo and Graham, 2014).

### 1.4. The current study

In two exploratory studies, we examined predictors of individual differences in early curiosity. In Study 1, we developed the Early Multidimensional Curiosity Scale (EMCS), to explore whether infants’ looking preferences for physically impossible events are associated with infants’ everyday curiosity and caregivers’ curiosity-promoting behaviors. In Study 2A, using a larger sample of children, we examined the underlying structure of the EMCS by conducting an Exploratory Factor Analysis. In Study 2B, we used the results from Study 2A to explore whether specific aspects of infant curiosity and caregiver curiosity-promoting behaviors predict individual differences in infants’ looking preferences for physically impossible events.

## 2. Study 1

### 2.1. Introduction

Using a violation-of-expectation paradigm, we explored whether infants’ looking preferences for physically impossible events are associated with their everyday curiosity and caregiver curiosity-promoting activities. We used violation-of-expectation tasks that violated infants’ understanding of continuity and solidity principles (i.e., relocation, occlusion, and solidity tasks) as these methods have been shown to have strong construct validity (Stahl and Kibbe, 2022). We tested children across infancy and early toddlerhood (10- to 24- months) because versions of this paradigm have been successfully used with infants (for a review see Baillargeon, 2004) and toddlers (e.g., 17- to 20-month-olds, Powell et al., 2018), and allows us to capture variability in individual differences in curiosity as those differences are first emerging. To control for other factors that might contribute to individual differences in infants’ looking patterns in violation-of-expectation tasks, we included measures of infants’ vocabulary (the MacArthur Communicative Development Inventory, MCDI; Fenson et al., 1994), temperament (i.e., effortful control, measured by the Early Childhood Behavior Questionnaire, ECBQ; Putnam et al., 2006), and caregiver trait curiosity (i.e., the Joyous Exploration subscale of the 5DC adult curiosity inventory; Kashdan et al., 2018).

If individual differences in infants’ visual preferences for physically impossible events relate to everyday curiosity and related experiences, their looking to physically impossible events (controlling for looking to possible events) should be predicted by caregiver reports of everyday curiosity and caregiver curiosity-promoting behaviors, but not by infant temperament or general, cognitive functioning (as measured by vocabulary size), or caregiver trait curiosity. Though vocabulary size is not a direct measure of infants’ general cognitive abilities, previous research has documented that vocabulary is predictive of cognitive functioning across domains, including those unrelated to language (i.e., working memory, spatial reasoning, pattern matching, learning, and problem-solving; Marchman and Fernald, 2008).

### 2.2. Methods

#### 2.2.1. Participants

Forty-seven full-term (i.e., born at or after 36 weeks), typically developing infants and toddlers (22 females, *M_age_* = 16.83 months, *SD_age_* = 4.31, *range* = 10.29–24.59 months) and their caregivers participated (43 mothers, 2 fathers, 2 did not complete the parent questionnaires, *M_age_* = 32.52 years, *SD_age_* = 3.25). Children were identified by their parents as White (70.2%), Asian/Asian American (10.6%), mixed race (10.6%), Black/African American (2.1%), Latinx/Hispanic/Latin American (2.1%), or did not report (4.3%). Caregivers reported that they had completed some college (4.4%), a 4-year college degree (40%), or a graduate school degree (55.6%).

Participants were recruited from a university-maintained database and websites such as Reddit, Children Helping Science,^1^ and social media. Our sample size was selected based on previous research examining individual differences in infant cognition using looking-based measures (e.g., Reuter et al., 2018; Perez and Feigenson, 2021). Families received a $15 gift card to a children’s bookstore and a digital Baby Scientist Certificate. Families also received a “Baby Scientist Kit” package in the mail that included small toys, printed experiment instructions, a family activity bundle, and a newsletter created by our lab. This study was approved by the ethics committee at Arizona State University (STUDY00012751). The methods reported here were performed in accordance with the guidelines and regulations outlined for this protocol.

Two caregivers did not complete the surveys. Data from infants of these two caregivers were excluded from analyses using survey data. Data from three infants were excluded from analyses using looking time data: one subject exhibited fussiness during all three videos (i.e., showed visible signs of distress while whining and refused to look at the display), one subject exhibited fussiness prior to viewing the videos (and therefore did not have an opportunity to view them), and one subject failed to meet the attentional criteria for all three videos.

Additional exclusions for looking time analyses were applied at the trial level: 24 of the 132 trials were excluded. Seven were excluded because infants failed to meet the attentional criteria in the familiarization or test phase (*n_Relocation_* = 3, *n_Occlusion_* = 1, *n_Solidity_* = 3). In the familiarization phase, this was defined as viewing at least 50% of the familiarization trial. In the test phase, this was defined as viewing the critical event (i.e., the moment at which the expected or unexpected event occurred). Fourteen trials were excluded because we were unable to compute a looking Preference Score (i.e., looking time in both test trials within a task was at ceiling, as in Scott and Schulz (2017); *n_Relocation_* = 7, *n_Occlusion_* = 2, *n_Solidity_* = 5), two trials were excluded due to a technical error which caused a video glitch during the testing phase (Relocation events), and one trial was excluded due to a Preference Score greater than 3 SD below the mean (Solidity event). Preference Scores were only calculated for a task if infants had valid looking time data for both the impossible and possible test events.

#### 2.2.2. Procedure

This study was conducted over Zoom during the COVID-19 pandemic. Prior to the study appointment, caregivers were emailed video instructions that outlined how to set up the virtual study session. Caregivers received a printed version of these instructions in the mail along with a study checklist to ensure they were fully prepared for the study. Participants were required to use a laptop or desktop computer, and were instructed to (as much as possible) set up a distraction-free environment. Infants were instructed to be seated in a child-friendly chair just out of reach of the computer. The average screen size was 14.76 inches (*SD* = 2.36, *range* = 10–21 inches). Screen size did not correlate with infants’ looking behaviors during the tasks (*r* = −0.08, *p* = 0.62).

At the time of the session, caregivers and participants met with an experimenter on Zoom who facilitated the session and sent caregivers a Qualtrics link that contained all study materials and video stimuli. Prior to participation, informed consent was obtained from a parent and/or legal guardian. Caregivers were instructed to remain neutral and look at the floor while the experimental videos were playing. In between experimental videos, caregivers were cued to look up at the screen, and progress to the next video. The data presented in the main text were drawn from a larger study investigating the development of active learning.

The study began with a one-minute warm-up video to acclimate infants to the testing environment, ensure the sound was played at the desired volume, and to provide feedback to the caregivers on the testing setup. If the infant was not in the optimal position for subsequent coding, or if the caregiver interfered with the infants’ viewing of the video, the experimenter intervened to provide feedback. After the one-minute warm-up video, infants watched a series of 4 videos (total duration of 7 min) before they were presented with the violation-of-expectation events described here. Caregivers were instructed to provide their infants with a break in between videos if needed. After completing the experimental session, caregivers were sent another link to a series of questionnaires on Qualtrics, a subset of which are described below.

#### 2.2.3. Violation-of-expectation tasks

Infants viewed three different physical events, each containing an initial familiarization phase (repeated twice) followed by two test events presented sequentially that contained either a possible or impossible outcome. All test events ended with a 10-s freeze-frame of the outcome, during which time infants’ cumulative looking at the screen was measured. Though violation-of-expectation paradigms typically use a 30–60 s coding window (Rubio-Fernández, 2019), others have used 10 s (e.g., Hood et al., 2003; Perez and Feigenson, 2022; Colomer and Woodward, 2023). We selected a relatively short window because pilot testing over Zoom revealed that infants were easily distracted by their home environment, so a short window was optimal for maintaining their attention across all trials.

**Figure 1:**
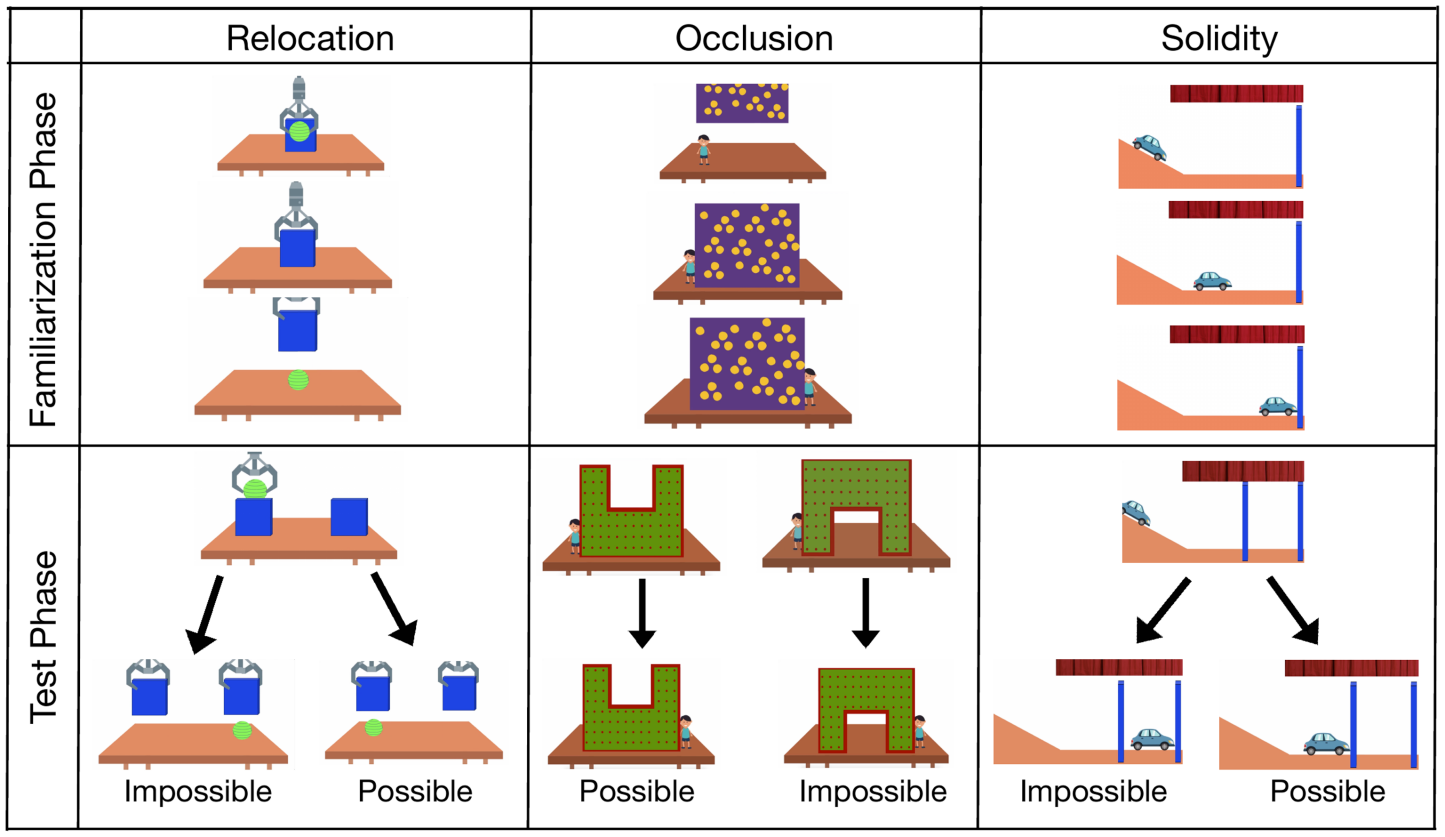
Sequence of still images of three different types of physical events shown in the violation-of-expectation tasks. In each event, infants were shown a familiarization phase (repeated twice) followed by two test events that contained either a possible or matched impossible outcome. All events in the test phase ended with a 10-s freeze-frame of the outcome event during which time infants’ cumulative looking at the screen was measured.

To calibrate infants’ gaze on the screen for subsequent coding, infants viewed an initial video that presented four looming stars in each corner of the screen presented sequentially. A brief attention-getter (a looming star in the center of the screen) was presented in between trials to capture infants’ attention. The order of the violation-of-expectation events was presented in a fixed order (i.e., Relocation, Occlusion, then Solidity) to prevent different orders from introducing another source of variance, and thereby allow us to capture individual differences uniquely explained by infants’ visual preferences for the presented stimuli (Goodhew and Edwards, 2019). The test events were presented in a pseudo-counterbalanced order: the impossible test event occurred first in the Relocation and Solidity events, and the possible test event occurred first in the Occlusion event (see Figure 1; Supplementary Video S1).

The violation-of-expectation tasks used here were not a direct replication of previous violation-of-expectation tasks. Traditionally, violation-of-expectation tasks are designed to detect group-level effects (i.e., an overall preference for impossible events), and are therefore constructed to generate high mean levels of looking to impossible events (in some cases resulting in ceiling effects), and little variability across infants (Byers-Heinlein et al., 2021). Here, we aimed to capture inter-subject variability in infants’ looking preferences by modifying a traditional design of the violation-of-expectation paradigm. While modifications were necessary because we collected data during the COVID-19 pandemic, it simultaneously contributed to our goal of increasing variability in infants’ looking preferences. Unlike traditional violation-of-expectation tasks conducted in controlled laboratory settings (e.g., a dark room with only video stimuli displayed), our task was conducted in children’s homes over Zoom. Exclusion due to infant distraction and distress tends to be higher in online testing compared to in the laboratory, suggesting it is more difficult to capture infants’ attention online, likely because there are more distractors competing for infants’ attention (Scott and Schulz, 2017; McElwain et al., 2021). In the current study, despite instructing caregivers to set up a minimally distracting environment, all families’ testing setups included myriad distractions (e.g., pets, siblings, etc.). Thus, longer looking to physically impossible events in our paradigm may not only represent increased interest in physically impossible events relative to possible events, but also relative to other interesting stimuli in infants’ environment.

##### 2.2.3.1. Relocation event

The Relocation familiarization event displayed a crane dropping a ball behind an opaque box. After dropping the ball, the crane lifted the box to reveal the hiding ball. In the test events, a second box appeared and the crane dropped the ball behind one of the two boxes. In the possible test event, the boxes were lifted to reveal the ball in its original position; in the impossible test event, the boxes were lifted to reveal the ball in a new position, as if it had magically transported itself.

##### 2.2.3.2. Occlusion event

The Occlusion familiarization event began with a doll standing beside an opaque, rectangular panel. A crane lifted the panel to reveal there was nothing hiding behind it. The panel returned to its original location and the doll moved behind the panel and appeared on its other side. The test events revealed the same doll standing beside a new panel that had a central portion removed. In the possible test event, the cut-out was located at the top of the panel, precluding infants from seeing actions occurring behind the panel. The doll moved from one side of the panel to the other. In the impossible test event, the cut-out was located at the bottom of the panel, granting infants visual access to actions occurring behind the panel. The doll moved from one side of the panel to the other but was not visible during the transition, as if it had momentarily disappeared.

##### 2.2.3.3. Solidity event

In the Solidity familiarization event, a car slid down a hill and stopped upon reaching a wall at the end of the hill. The test events included another wall located a short distance past the established wall. As the car descended down the hill in the test events, a curtain was lowered to hide the event outcome. In the possible test event, the curtain lifted to reveal the car stopped in front of the first wall; in the impossible test event, the curtain lifted to reveal the car stopped in front of the second wall, as if it had magically moved through the first wall.

#### 2.2.4. Coding and reliability

Infants’ visual fixation during test events (i.e., the 10 s freeze-frame following the physically possible and impossible events) was coded offline using DataVyu software.^2^ At each 33 ms block of time, the primary coder identified whether infants were visually fixated on the screen or not. Twenty percent of videos were re-coded offline to establish high inter-rater reliability (intra-class correlation = 0.97; 95% CI = 0.95–0.98; *p* < 0.001). Due to restrictions placed on our lab during the COVID-19 pandemic, the primary coder was also the primary experimenter and was therefore not naive to experimental conditions (i.e., which test trial was the impossible versus possible event). However, the secondary reliability coder was naive to experimental conditions and was unaware of which test trial was impossible versus possible.

#### 2.2.5. Parent questionnaire

##### 2.2.5.1. The Early Multidimensional Curiosity Scale (EMCS): A comprehensive questionnaire designed to measure curiosity and related experiences during infancy and childhood

The EMCS is a 55-item scale we created to assess curiosity across a diverse range of settings in 10- to 78-month-olds. The scale includes questions about child curiosity (22 items) and questions about caregiver curiosity-promoting behaviors (33 items). Together, these measures were designed to capture a holistic picture of children’s early curiosity and related experiences. An overall curiosity score, the “EMCS total,” was computed by averaging all 55 items together. The alpha coefficient of the overall scale was.77 (95% CI = 0.67–0.86). All items in the scale are on a 5-point scale, ranging from “never” to “always,” “strongly disagree” to “strongly agree,” “extremely dislike” to “extremely like.” The full scale is presented in Appendix A of the Supplementary materials.

##### 2.2.5.2. Caregivers’ preferences for curiosity-themed books and toys

To overcome ecological validity challenges inherent in asking caregivers to reflect on their attitudes, beliefs, and everyday behaviors, we created two measures that aimed to capture caregivers’ preferences for curiosity-themed books and toys. These measures aimed to more accurately capture caregivers’ real-world curiosity-stimulating behaviors and preferences by asking what types of books and toys they would choose to buy for their children. Caregivers were told that our lab would soon be purchasing new books and toys and would like their input on which to select (they did not receive copies of these books or toys, they only read about them). To measure caregivers’ preferences for curiosity-themed books, caregivers read vignettes that described three different books, and were prompted to select which one they would choose if they were buying one for their child. Each book had a single distinctive theme that centered around teaching children either curiosity, prosociality, or counting. The vignettes were created to be matched on all dimensions (e.g., length, overall engagement) aside from the virtue conveyed to children. Likewise, to measure caregivers’ preferences for curiosity-themed toys, caregivers were given three descriptions of toys and asked to select one they would choose for their child. The toy themes centered around either curiosity, animals, or music. These new measures allowed us to capture an ecologically-relevant assessment of the degree to which parents chose to teach their children about curiosity as a virtue, as opposed to other important developmental themes. These measures are presented in Appendix B in Supplementary materials.

##### 2.2.5.3. The I/D-Type young children curiosity scale (I/D-YC)

The I/D-YC is a 10-item survey measuring two types of children’s curiosity (Piotrowski et al., 2014): the Interest Type (I-Type) subscale measures epistemic curiosity, or the intrinsic joy in discovering new knowledge (e.g., “My child shows visible enjoyment when discovering something new”), and the Deprivation Type (D-Type) subscale measures children’s drive to reduce uncertainty and fill uncomfortable gaps in knowledge (e.g., “My child devotes considerable effort trying to figure out things that are confusing or unclear”). Caregivers rated each question on a 4-point scale ranging from “almost never” to “almost always.” Responses were averaged within each subscale section to calculate an I-Type and D-Type score for each participant. Though this scale was validated with older children (3- to 8-year olds) and therefore contained several age-inappropriate questions for infants (e.g., “My child enjoys talking about things that are new to him/her”), we administered it to measure the convergent validity of the EMCS, because it is the only validated survey measure of curiosity during childhood.

##### 2.2.5.4. The five-dimensional adult curiosity scale (5DC)

The 5DC measured caregivers’ self-report of their own trait curiosity level. This scale includes five distinct aspects of adult curiosity (Kashdan et al., 2018): Joyous Exploration, Deprivation Sensitivity, Stress Tolerance, Social Curiosity, and Thrill Seeking. The analyses presented here include only the Joyous Exploration subscale (i.e., the enjoyment in learning new information), because it is most conceptually related to the epistemic curiosity we were interested in the current work (example item: “I find it fascinating to learn new information,” see also Kashdan et al., 2020). Responses were on a 7-point scale ranging from “Does not describe me at all” to “Completely describes me”.

##### 2.2.5.5. Macarthur communicative development inventory (MCDI)

The MCDI is an 89-item vocabulary checklist designed to measure children’s receptive and productive vocabulary development (Fenson et al., 1994). Caregivers reported whether their children “understands” or “understands and says” each item. The data presented in the current study are the sum total from the “understand and says” category.

##### 2.2.5.6. The early childhood behavior questionnaire (ECBQ)

The ECBQ is a 36-item survey designed to measure temperament in children aged 1 to 3 years (Putnam et al., 2006). Three subscales include: Surgency, Negative Affectivity, and Effortful Control. Caregivers rated each question on a 7-point scale ranging from “Never” to “Always.” The analyses presented here include only the Effortful Control subscale (e.g., “When s/he was upset, how often did your child become easily soothed?”) because previous research has shown that this dimension of temperament is strongly linked to key components of curiosity, such as flexible attention shifting, persistence, and positive classroom functioning later in childhood (Simonds et al., 2007; Valiente et al., 2010).

### 2.3. Results

#### 2.3.1. Infants’ looking preferences for impossible events

Analyses were performed in R (v4.1.2; R Core Team, 2021) using the functions *lm* and *cor.test*. Since our central question was about individual differences in looking preferences for impossible versus possible events, we calculated a “Preference Score” for each infant by taking the difference between their looking time to the impossible versus possible event in each trial rather than simply comparing their raw looking time to impossible events to possible events. The Preference Score indicates the extent to which infants attended to impossible events, controlling for their attention to possible events. Since the Preference Score captures infants’ selective attention, not just their general level of visual attention, it is a more precise indicator of infants’ visual preferences for impossible events.

Though our study was not designed to directly replicate the group-level effect of infants’ looking preference for impossible events over possible events (Perez and Feigenson, 2021), we first asked whether infants showed a significant preference for looking toward impossible events across the three types of violation events. Infants showed a marginally significant preference for the impossible events compared to the matched possible events (*M =* 0.33 s, *SD* = 1.29, *t*(43) = 1.67, *p* = 0.051). Preference Scores did not differ significantly from zero in the Relocation (*M* = 0.06 s, *SD* = 2.33, *t* (31) = 0.14, *p* = 0.445) or Occlusion event (*M* = −0.27 s, *SD* = 1.83, *t* (40) = −0.95, *p* = 0.826), but did differ from zero in the Solidity event (*M* = 1.34 s, *SD* = 2.13, *t* (34) = 3.71, *p* < 0.001; see Supplemental Materials for a more detailed breakdown of infants’ looking time data). Because these analyses were conceptual replications of prior research, one-tailed tests were used.

To capture infants’ overall preference for impossible events across different event types, we calculated an average Preference Score for each infant by averaging their Preference Scores across trials. If infants were missing a Preference Score for one of their three trials, we computed their average Preference Score using their existing trial data (e.g., averaging two trials instead of three if one trial was unusable).

**Figure 2:**
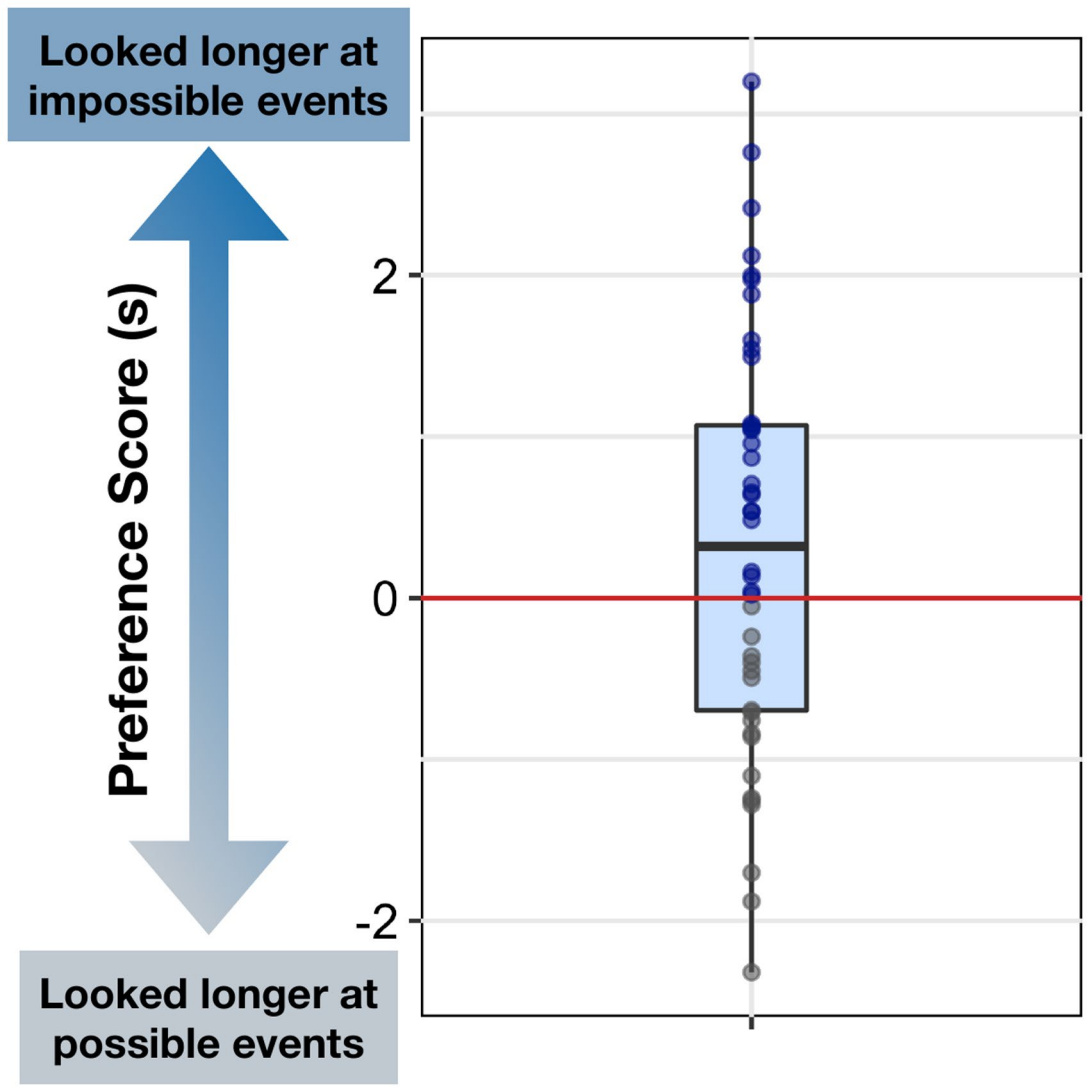
Individual differences in infants’ Preference Score. The y-axis represents infants’ Preference Score for the impossible events, calculated by subtracting the looking time at possible events from the looking time at the matching impossible events. Preference Scores higher than 0 = looked longer at impossible events (blue dots); Preference Scores lower than 0 = looked longer at possible events (gray dots).

Though not all infants showed a significant preference for impossible events, importantly for our central question, there were high degrees of individual variability in infants’ average Preference Score, with a minimum score of −2.32 s and a maximum of 3.2 s (see Figure 2). This variability across participants allowed us to examine various factors that may be correlated with infants’ looking preferences for physically impossible events. Critically, there was no correlation between age and average Preference Score (*r =* −0.005, *p = 0*.977), suggesting that the paradigm was equally appropriate across the entire age range tested.

#### 2.3.2. Predicting variability in infants’ looking preferences for impossible events

Two multiple regressions were conducted to explore predictors of infants’ looking preferences for impossible events. We, first, included the following predictors in a single regression model predicting infants’ looking preferences: the Early Multidimensional Curiosity Scale (EMCS) total score (i.e., infants’ everyday curiosity and related experiences provided by caregivers), caregivers’ selection of curiosity-themed books and toys, caregivers’ own curiosity (Joyous Exploration; 5DC), infants’ vocabulary size (MCDI), and infants’ temperament (Effortful Control; ECBQ). Then, in a second regression model, we separated the EMCS into two parts (child curiosity measures and caregiver activity measures) and included two separate scores instead of the EMCS total score in the second regression model to explore which aspect of the EMCS (child’s own curiosity or caregiver curiosity activities) predicts infants’ looking preferences.

**Figure 3:**
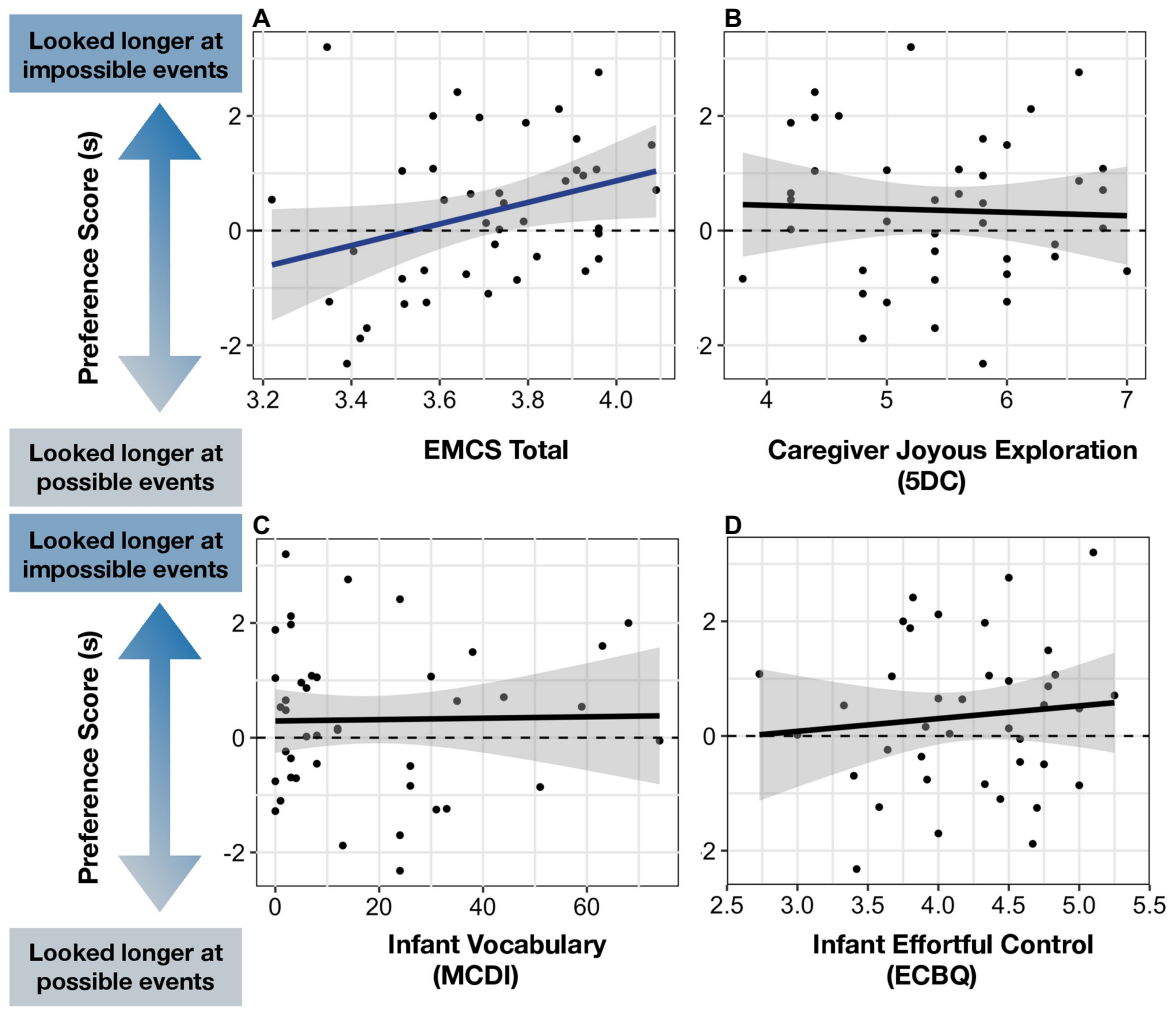
Relations between four predictors and infants’ Preference Score: **(A)** EMCS Total (Early Multidimensional Curiosity Scale), **(B)** Caregiver Joyous Exploration (5DC), **(C)** Infant Vocabulary (MCDI), **(D)** Infant Effortful Control (ECBQ).

**Figure 4:**
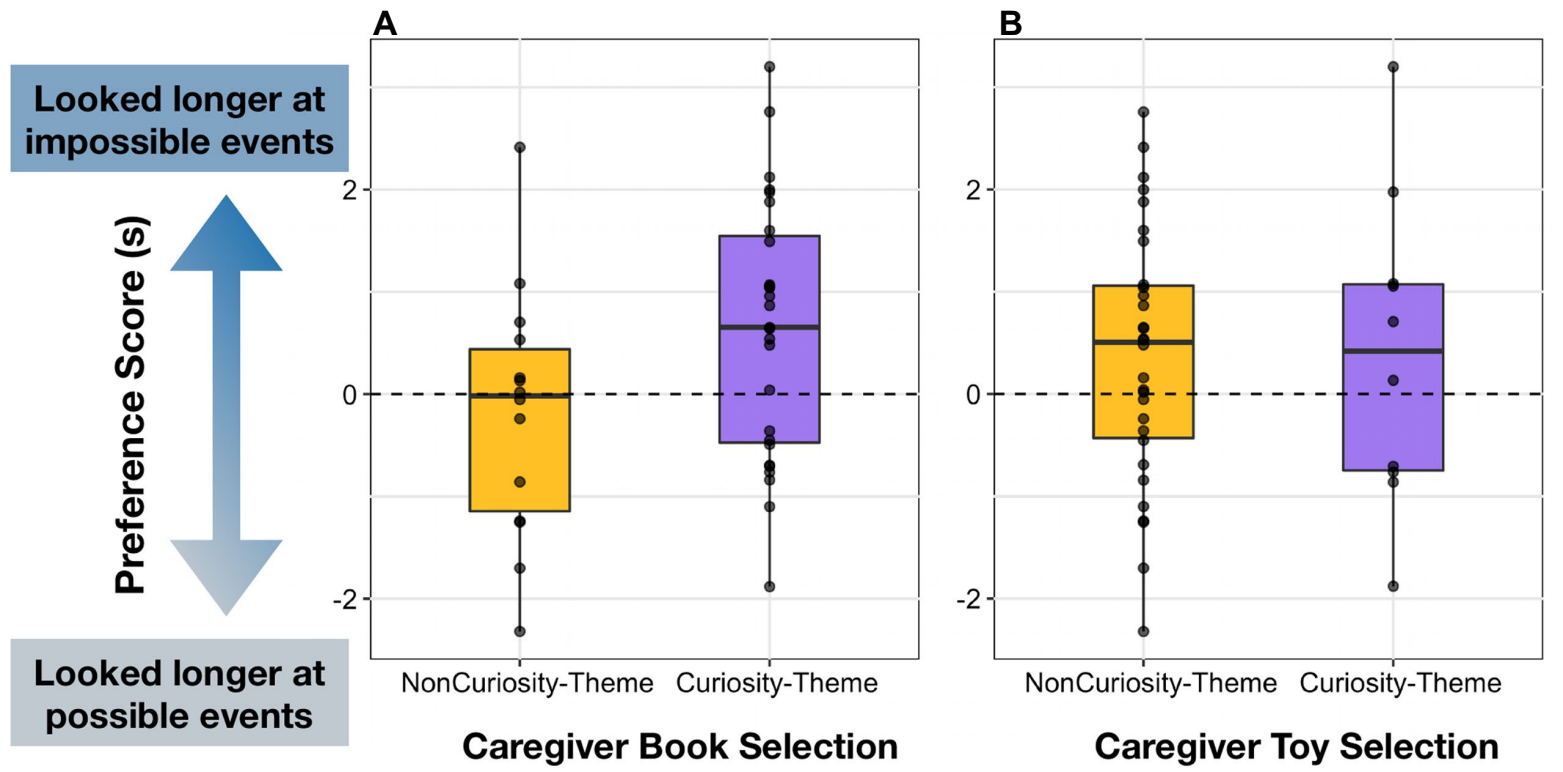
Infants’ Preference Score based on caregivers’ curiosity-themed book and toy selection for their child. In the Book Selection, parents were given three options: curiosity, prosociality, or counting. Prosociality and counting books were coded as a non-curiosity-themed book. In the Toy Selection, participants were given three options: curiosity, animal, or music. Animal and music toys were coded as a non-curiosity-themed book.

Descriptive statistics and correlations among main variables for both regression models are presented in Table 1. Infants’ preferences for impossible events were significantly predicted by the EMCS total score, such that infants who showed higher everyday curiosity and experienced more curiosity-promoting activities provided by parents tended to look longer at physically impossible events compared to infants with lower scores on the EMCS total (*B* = 2.49, *p* = 0.032; see Figure 3). Infants’ Preference Score was marginally predicted by caregivers’ selection of curiosity-themed books, such that caregivers who chose curiosity-themed books had infants who showed marginally higher looking preferences for physically impossible events (*B = 0*.95, *p* = 0.066; see Figure 4). Caregivers’ selection of curiosity-themed toys, caregiver trait curiosity, infants’ vocabulary size, and infants’ temperament did not significantly predict infants’ Preference Score (all *p’*s > 0.1, see Table 2).

**Table 1:** Descriptive statistics and correlations among the main variables.

Measure	M (SD)	1	2	3	4	5	6	7
1. Preference Score	0.33 (1.29)	-						
2. EMCS Total	3.70 (0.21)	0.31*	-					
3. EMCS Child Curiosity	3.65 (0.26)	0.30^†^	0.80***	-				
4. EMCS Caregiver Activity	3.75 (0.27)	0.19	0.81***	0.29^†^	-			
5. Caregiver Joyous Exploration (5DC)	5.43 (0.85)	−0.04	0.46**	0.14	0.60***	-		
6. Infant Vocabulary (MCDI)	17.31(20.44)	0.02	0.09	0.04	0.1	−0.03	-	
7. Infant Effortful Control (ECBQ)	4.22 (0.71)	0.1	0.38**	0.19	0.42**	0.30*	0.35*	-

EMCS (Early Multidimensional Curiosity Scale), EMCS Child Curiosity (average score of child curiosity items), EMCS caregiver activity (average score of parent curiosity-promoting activity items), caregiver curiosity trait measure (5DC), infant vocabulary measure (MCDI), infant temperament measure (ECBQ). ^†^*p* ≤ 0.10, *p ≤ 0.05, **p ≤ 0.01; ***p ≤ 0.001.

**Table 2:** Multiple regression analysis summary of infants’ average Preference Score.

Predictors	*B*	*β*	*t*	*p*	95% CI for *B*
EMCS Total	2.49*	0.41	2.24	0.032	[0.23, 4.76]
Curiosity-themed book selection	0.95^†^	0.35	1.9	0.066	[−0.07, 1.97]
Curiosity-themed toy selection	0.34	0.11	0.67	0.507	[−0.69, 1.37]
Caregiver joyous exploration (5DC)	−0.23	−0.15	−0.85	0.404	[−0.78, 0.32]
Infant vocabulary (MCDI)	0.01	0.12	0.66	0.517	[−0.02, 0.03]
Infant effortful control (ECBQ)	−0.38	−0.19	−0.91	0.368	[−1.24, 0.47]

EMCS (Early Multidimensional Curiosity Scale), caregiver curiosity trait measure (5DC), infant vocabulary measure (MCDI), infant temperament measure (ECBQ). ^†^*p* ≤ 0.10 and *p ≤ 0.05.

We conducted an additional exploratory analysis to examine whether the child curiosity measures (22 items) and caregiver activity measures (33 items) on the EMCS *independently* predicted infants’ looking preference score, we divided the EMCS into two subscales: the Child Curiosity Subscale (including only items that asked about *children’s* curiosity) and the Caregiver Activity Subscale (including only items that asked about the *caregivers’ activities*). We then ran the same regression model presented earlier, but with two separate scores from each subscale. Infants’ Preference Score was not significantly predicted by the Child Curiosity or the Caregiver Activity Subscales alone, despite a positive trend (*B* = 1.13, *p* = 0.188 and *B* = 1.41, *p* = 0.185, respectively). Type I error corrections were not applied because these analyses were exploratory (Bender and Lange, 2001).

#### 2.3.3. The Early Multidimensional Curiosity Scale (EMCS) convergent validity

To test the convergent validity of the child curiosity items on the EMCS, we tested whether the average score of all items on the Child Curiosity Subscale correlated with an established measure of young children’s epistemic curiosity, the average score of all items on the I/D-Type Young Children Curiosity Scale (I/D-YC; Piotrowski et al., 2014). Results revealed that scores on these two scales were significantly correlated (*r* = 0.33, *p* = 0.027). We also tested the convergent validity of the caregiver curiosity-promoting activities items on the EMCS by examining whether the Caregiver Activity Subscale was positively correlated with a validated measure of adult trait curiosity (Joyous Exploration subscale on the 5DC Scale; Kashdan et al., 2018). Results showed that scores on these two scales were significantly correlated (*r* = 0.60, *p* < 0.001). Together, these results provide evidence for the convergent validity of the EMCS.

To ensure that the EMCS scale was equally appropriate across the age range tested, we examined the distribution of the EMCS total score across ages. There was no significant relation between infants’ age and the EMCS total score (*r* = 0.15, *p* = 0.330). This was true when examining the relations between age and the Child Curiosity Subscale and the Caregiver Activity Subscale (*r* = 0.08, *p* = 0.627 and *r* = 0.15, *p* = 0.326, respectively).

### 2.4. Discussion

Study 1 examined predictors of individual differences in infants’ visual preferences for physically impossible events. Results revealed that infants’ everyday curious behaviors and curiosity-promoting experiences from caregivers *together* predicted their looking preferences for physically impossible events over closely matched possible events. Infants’ looking preferences were not predicted by infants’ temperament traits, vocabulary size, or caregivers’ own trait curiosity. These findings suggest that individual differences in infants’ preferences for physically impossible events may reflect an early form of curiosity, but not temperament traits, general cognitive functioning, or overall information-processing abilities.

A follow-up analysis tested the unique contributions of the Child Curiosity and Caregiver Activity Subscales of the EMCS on infants’ Preference Score. Though both measures were positively related to infants’ Preference Score, each measure alone did not reach statistical significance. Critically, however, the EMCS was designed to capture “diverse aspects” of infants’ curiosity and caregivers’ curiosity-promoting behaviors, not just sheer levels of infant curiosity or frequency of caregiver curiosity-promoting behaviors. Thus, in Study 2A, we analyzed the factor structure of the Child Curiosity Subscale and Caregiver Activity Subscale by assessing whether certain items within each set can be organized into meaningful subgroups, which may reflect the diverse spectrum of infants’ curiosity and caregiver curiosity-promoting behaviors. In Study 2B, we use results from Study 2A to test whether specific aspects of early curiosity and caregiver activities predict infants’ visual preferences for physically impossible events.

## 3. Study 2A

### 3.1. Introduction

In Study 1, we described the development of the Early Multidimensional Curiosity Scale (EMCS), and investigated whether infants’ looking preferences for physically impossible events were predicted by EMCS scores. Treating child curiosity and caregiver activities as a singular measure (i.e., simply calculating an average score for each subscale) as we did in Study 1 may not have allowed us to detect whether specific aspects of curiosity and/or caregiver curiosity-promoting activities uniquely relate to infants’ looking preferences for physically impossible events.

In Study 2A, we explored the underlying factor structure of two subscales of EMCS using an exploratory factor analysis (EFA). The EFA allowed us to test whether children’s early curiosity consists of multiple, independent dimensions, and relatedly, whether there are diverse ways caregivers promote children’s curiosity. Identifying the dimensionality of each subscale of the EMCS is critical for understanding the nature of early curiosity and curiosity-promoting activities. Since the study was exploratory, we did not set strong hypotheses about the specific number of factors included within each subscale.

Studies on older children and adults have revealed that curiosity is a multidimensional construct composed of distinct aspects of curiosity (Kashdan et al., 2018). It is possible that curiosity earlier in development has a similar nature, such that early curiosity is expressed in different ways and can be captured by distinct underlying factors. For example, questions that tap into interests in learning about new people may be related to each other, but distinct from questions that capture interests in exploring the physical environment. Alternatively, it may be more appropriate to conceptualize curiosity in early childhood along a single continuum, such that individuals either score high or low on all questions related to early curiosity—suggesting that it is only through various experiences combined with cognitive maturation that early curiosity becomes more differentiated and adultlike.

Research has also yet to examine the underlying factor structure of caregiver curiosity-promoting activities. One possibility is that curiosity-promoting activities, similar to trait curiosity, consist of multiple dimensions. A multidimensional factor structure would suggest that caregivers’ have unique ways of encouraging curiosity in young children such that different subtypes of curiosity-promoting behaviors cluster together and are distinct from others. For instance, promoting curiosity in young children by exposing them to novelty may be distinct from boosting curiosity by taking them to an awe-inducing experience, such as a nature walk. Caregivers may tailor their behaviors to the unique way their children express curiosity, or they may have their own way of promoting curiosity based on their own interests or personality. Alternatively, caregiver curiosity-promoting activities may have a unidimensional factor structure. If so, caregivers will either score “high” or “low” in terms of the sheer amount of curiosity-promoting activities they tend to engage in for their infants in everyday life.

### 3.2. Methods

#### 3.2.1. Participants

Two hundred and eleven caregivers (198 mothers and 13 fathers, *M_age_* = 35.6 years, *SD_age_* = 4.91) with a child between the ages of 10 and 78 months from two independent samples participated. Caregivers identified themselves as White (70.5%), Asian/Asian American (16.7%), Latinx/Hispanic/Latin American (7.1%), mixed race (2.4%), Native American/American Indian/Alaskan Native (1.9%), Black/African American (0.5%), Middle Eastern/Middle Eastern American (0.5%), or did not report (0.5%). The majority of caregivers had a high education level: completed high school (2.8%), some college (9%), a 4-year college degree (38.4%), or a graduate school degree (49.8%).

The infant sample (*N* = 54, 26 females, 27 males, and 1 non-binary, *M_age_* = 16.55 months, *SD_age_* = 4.51, *range* = 10.29–24.59 months) included participants from Study 1 who had complete questionnaire data (*N* = 45) and an additional set of infants who were recruited for Study 1, but did not have looking time data because they were pilot participants for the Zoom experimental testing setup (*n* = 5) or were born prematurely (*n* = 4). The child sample (*N* = 157, 77 females, 79 males, 1 non-binary *M_age_* = 58.33 months, *SD_age_* = 10.63, *range* = 41.0–78.97 months) was independently recruited for a study on childhood exploration and decision-making. The child sample received a child version of the EMCS and was included here to reach the recommended sample size for an exploratory factor analysis (Worthington and Whittaker, 2006).

The child sample was recruited using the same method as described in Study 1. Because items on the scales were highly similar across ages, all analyses include both age groups to ensure sufficient statistical power and to test the underlying structure of the EMCS across a wider period of development. This study was approved by the ethics committee at Arizona State University (STUDY00012799).

#### 3.2.2. Procedure and measures

The procedure for administering the Early Multidimensional Curiosity Scale (EMCS) was identical to Study 1. After children participated in a live Zoom session with an experimenter, caregivers completed an online Qualtrics survey consisting of self-report questionnaires and demographic questions.

The child version of the EMCS (for use with children 2 years and older) was highly similar to the infant version of the EMCS (for use with children younger than 2 years), with the exception that three items were adapted to ensure age appropriateness. These three items had a direct equivalent across versions (e.g., gesturing/vocalizing was changed to talking/question-asking across versions), and therefore were included in the factor analysis. There were an additional nine items that were not included in the analysis either because they did not have direct equivalents across versions (*N =* 6 items) or because they were added for the child version only (*N* = 3 items). The full scale is presented in Appendix A of the Supplementary materials.

In sum, the factor analysis on the Child Curiosity Subscale included only the 16 items that had equivalents across both the infant and child version. The alpha for the Child Curiosity Subscale was 0.70 (95% CI = 0.64–76). Items on the Caregiver Activity Subscale were exactly the same across both versions, and so all 33 items were included in the factor analysis. The alpha for the Caregiver Activity Subscale was 0.84 (95% CI = 0.81–87).

### 3.3. Results

#### 3.3.1. Factor structure of the Child Curiosity Subscale

##### 3.3.1.1. Exploratory factor analysis

Analyses were performed in R using *fa* function (psych R package). Bartlett’s test of sphericity reached statistical significance (*χ*^2^ = 788.96, *df* = 120, *p* < 0.001) and the Kaiser–Meyer–Olkin measure of sampling adequacy (KMO) was 0.65, indicating that the data were suitable for an exploratory factor analysis (Kaiser, 1974). Next, parallel analysis (Horn, 1965) and visual inspection of the scree plot (Cattell, 1966) were conducted to determine the number of factors to extract. We elected to use parallel analysis and scree plot inspection over the “Kaiser rule” method because although the Kaiser rule is commonly used, it has been demonstrated to perform poorly compared to parallel analysis (Fabrigar et al., 1999; Garrido et al., 2016). Parallel analysis and visual inspection of the scree plot supported a four-factor solution. Then, a principal axis factoring with a promax was run, specifying a four-factor solution with the 16 items. A promax oblique rotation was selected based on the theoretical assumption that the factors are related to one another. The four-factor solution explained 39.2% of the total variance. Five items that did not load on any factor (factor loadings ≤ |0.35|) were dropped. Then, the same extraction and rotation methods were performed on the remaining 11 items. A four-factor solution was supported again, which accounted for 51.6% of the total variance: Factor 1 accounted for 20.2%, Factor 2 accounted for 12%, Factor 3 accounted for 11.1%, and Factor 4 accounted for 8.2% (Table 3).

**Table 3:** Factor loadings for the final 11 items of the Child Curiosity Subscale.

Items	EFA				SocialCuriosity	Broad Exploration	Persistence	Information-Seeking
When visiting a new environment (such as a playground), how often does your child quickly gravitate toward other children?	**0.83**	0.08	0.08	−0.15
To what extent does your child enjoy interacting with new children?	**0.83**	−0.12	0.03	−0.01
When visiting a new environment (such as a friend’s home) how often does your child immediately go to where there are many people (versus more isolated areas)?	**0.79**	0.11	−0.01	−0.04
To what extent does your child enjoy interacting with new adults?	**0.42**	−0.06	−0.04	0.28
How likely is your child to explore the majority of toys in the room?	0.08	**0.9**	0.04	−0.03
How likely is your child to spend the whole time playing with one specific toy? [*R*]	−0.04	**0.67**	−0.06	0.02
When your child has difficulty completing a task, to what extent are they highly motivated to keep trying?	−0.05	0.01	**0.93**	−0.01
When your child has difficulty completing a task, to what extent do they give up immediately and shift their attention to another task? [*R*]	0.1	−0.04	**0.55**	0.01
How often does your child point, gesture, or talk about things that are new to them?	−0.04	−0.12	−0.04	**0.63**
When presented with unfamiliar objects, how often does your child point, gesture, or talk about things that are new to them?	0.11	0.11	−0.07	**0.52**
When something contradicts what your child knows about the world, how often does your child further explore, as if to figure out what happened?	−0.1	0.08	0.16	**0.42**

Items are reduced for the sake of clarity. See Supplementary materials for the original items. Items with [*R*] are reverse-coded items.

The pattern of factor loadings reflected conceptually meaningful and distinct groupings (indicated in bold in Table 3). We created a label for each factor that represented the underlying theme of all items within a factor, with a particular focus on items that loaded most highly on that factor (see Table 4). Factor 1, Social Curiosity, consisted of four items centered around children’s interest toward other people. Factor 2, Broad Exploration, consisted of two items measuring children’s preferences to explore and play with a wide range of toys (versus fixating on a select few toys). Factor 3, Persistence, consisted of two items asking about children’s tendency to persistently work to overcome challenges. Factor 4, Information-Seeking, consisted of three items asking about the degree to which children actively request and search for new information.

**Table 4:** Factor loadings for the final 19 items of the Caregiver Activity Subscale.

Items	EFA				
	Flexible Problem-Solving	Cognitive Stimulation	Diverse Daily Activities	Child-Directed Play	Awe-Inducing Activities
How much do you agree?: Encourage my child to look at problems from multiple perspectives.	**0.82**	0.17	−0.17	−0.05	−0.11
How much do you agree?: Encourage my child to embrace uncertainty.	**0.59**	0.03	−0.07	−0.02	−0.06
How often do you encourage your child to figure out how new things work on their own?	**0.53**	−0.08	0.03	−0.02	−0.05
How often do you ask your child open-ended questions?	**0.5**	0.08	−0.01	0.01	0.16
How frequently do you introduce your child to new cultures or languages?	**0.43**	−0.18	0.28	0.09	0.04
How much do you agree?: I encourage my child to participate in creative activities (such as pretend-play)	0.04	**0.61**	0.04	0.03	0.01
How much do you agree?: I allow my child to try new ways of playing with things, even when it may not be what those items were designed for.	−0.09	**0.6**	0.1	−0.06	−0.07
How much do you agree?: I encourage my child to try doing new things they have never done before.	−0.02	**0.53**	−0.1	0.07	0.04
How much do you agree?: I want to encourage my child’s interests, even if I do not share those interests.	0.01	**0.47**	−0.04	0.12	0.06
How frequently do you play with your child, using objects or toys for something outside of their intended purpose (such as wearing a pot like a hat)?	0.04	**0.44**	0.27	−0.22	−0.07
How much do you agree?: I encourage my child to use their imagination	0.29	**0.43**	0.05	0.06	0.03
How frequently do you introduce your child to unfamiliar objects (like whisks) or new toys?	−0.15	0.18	**0.67**	0.12	−0.01
When you do your hobbies, how often do you show your child what you are doing?	−0.02	−0.04	**0.52**	−0.1	−0.03
How frequently do you show your child different types of activities (such as cooking, music)?	0.21	0.01	**0.46**	0.06	0.1
How often does your child lead playtime?	0.01	0.04	0.09	**0.73**	−0.09
How often do you lead playtime? [*R*]	−0.03	0.06	−0.08	**0.64**	−0.02
How interested would you be in taking your child to a museum (such as a science museum)?	0	0.18	−0.16	−0.13	**0.82**
How interested would you be in taking your child to a live performance (such as a concert)?	0.07	−0.05	0	−0.04	**0.4**
How interested would you be in taking your child on a nature walk or hike with you?	−0.13	−0.03	0.08	0.03	**0.4**

Items are reduced for the sake of clarity. See Supplementary materials for the original items. Items with [*R*] are reverse-coded items.

##### 3.3.1.2. Confirmatory factor analysis

Next, we conducted a confirmatory factor analysis to test whether the four factors are clearly identifiable constructs as measured by the items they contain and fit our data. Model fit is considered good if the Comparative Fit Index (CFI) and the Tucker–Lewis Index (TLI) are greater than or equal to 0.95 (adequate if greater than or equal to 0.90), the Root-Mean-Square Error of Approximation (RMSEA) is less than or equal to 0.06 (adequate if greater than or equal to 0.08), the Standardized Root-Mean-Square Residual (SRMR) is less than or equal to.08 (Hu and Bentler, 1998, 1999), and *χ*^2^/*df* ratio is 3 or less (Hoyle, 2012). The results supported the structure from EFA by demonstrating that four factors, comprising all the 11 items, yielded adequate to good fit across multiple indices (CFI = 0.90; TLI = 0.86; RMSEA = 0.09; SRMR = 0.06; *χ*^2^/*df* = 2.5).

#### 3.3.2. Factor structure of the Caregiver Activity Subscale

##### 3.3.2.1. Exploratory factor analysis

We followed the same statistical approach to analyze the factor structure of the Caregiver Activity Subscale. Bartlett’s test of sphericity reached statistical significance (*χ*^2^ = 1,810.15, *df* = 528, *p* < 0.001) and Kaiser–Meyer–Olkin measure of sampling adequacy (KMO) was 0.75, showing that the data were suitable for EFA (Kaiser, 1974). A parallel analysis (Horn, 1965) and visual inspection of the scree plot (Cattell, 1966) supported a five-factor solution for the 33 items. A principal axis factoring with a promax rotation specifying a five-factor solution was conducted. The five-factor solution accounted for 31.2% of the total variance. Items that did not load on any factor (factor loadings ≤ |0.35|) or cross-loaded (factor loadings > |0.35| on more than one factor) were dropped. This iterative process eliminated a total of 14 items (12 non-loaded items and 2 cross-loaded items). The final round of EFA with the final 19 items revealed a five-factor solution, which explained 37.8% of the total variance: Factor 1 accounted for 10.4%, Factor 2 accounted for 10.1%, Factor 3 accounted for 6.2%, Factor 4 accounted for 5.6%, Factor 5 accounted for 5.5%.

The pattern of factor loadings reflected conceptually cohesive and unique groupings (indicated in bold in Table 4). We labeled each factor by considering the underlying theme of each item under the same factor, but mainly based on its highly loaded items (see Table 5). Factor 1, Flexible Problem-Solving, consisted of five items measuring the degree to which caregivers encourage children to be flexible in problem-solving by taking new perspectives (e.g., encouraging to look at problems from multiple perspectives), embracing uncertainty (e.g., asking open-ended questions), or overcoming challenges (e.g., encouraging to figure out problems on their own). Factor 2, Cognitive Stimulation, consisted of six items measuring the degree to which caregivers explicitly teach their children to think outside of the box (e.g., showing how to use objects or toys for something outside their intended purpose, such as playing with a familiar toy in a new way) or provide experiences that stimulate children’s imagination (e.g., pretend-play). Factor 3, Diverse Daily Activities, consisted of three items measuring the degree to which caregivers involve their children in a diversity of everyday activities and routines (e.g., introducing unfamiliar objects or different types of activities such as cooking, music). Factor 4, Child-Directed Play, consisted of two items measuring caregivers’ encouragement of their children’s lead in play (e.g., encouraging a child to take initiative in playtime). Factor 5, Awe-Inducing Activities, consisted of three items that measured caregivers’ interest in introducing their children to awe-inspiring experiences (e.g., going to a museum, live performance, and nature walk).

**Table 5:** Multiple regression analysis summary of Child Curiosity Subscale model.

Predictors	*B*	*β*	*t*	*p*	95% CI for *B*
Social Curiosity	−0.17	−0.1	−0.67	0.509	[−0.69, 0.35]
Broad Exploration	0.73*	0.39	2.67	0.011	[0.18, 1.29]
Persistence	−0.22	−0.1	−0.65	0.523	[−0.93, 0.48]
Information-Seeking	0.53	0.22	1.49	0.146	[−0.19, 1.25]

*p ≤ 0.05.

##### 3.3.2.2. Confirmatory factor analysis

We fit our data to the five-factor structure of Caregiver Activity Subscale gained from EFA following the same procedure used for the CFA in the Child Curiosity Model. The results revealed that the five-factor structure identified by EFA showed moderate to good fit (CFI = 0.93; TLI = 0.92; RMSEA = 0.04; SRMR = 0.06; *χ*^2^/*df* = 1.32).

### 3.4. Discussion

The results from Study 2A demonstrate that children’s early curiosity, as measured by parent report, is a multidimensional construct, composed of four distinct factors. These results also show that caregivers’ engagement in curiosity-promoting activities is similarly multifaceted, and comprised five distinct underlying factors, each of which may relate to infant curiosity in unique ways.

The four factors that comprise the Child Curiosity Subscale show close conceptual overlap with factors in curiosity scales used with older children and adults (Piotrowski et al., 2014; Kashdan et al., 2018). Social Curiosity (i.e., interest toward other people) has consistently been shown to be a unique aspect of curiosity in children and adults (Post and Walma van der Molen, 2019; Wagstaff et al., 2021), and has direct overlap with items identified in our Social Curiosity factor. Broad Exploration is similar to what Litman (2008) coined as “Interest Type” curiosity in children, and what Kashdan et al. (2018) termed “Joyous Exploration” in adults, both of which are related to curiosity motivated by the joy of new discoveries. Persistence captures children’s motivation to work hard to solve problems, and is in line with Kashdan and Steger’s (2007) finding that curiosity and persistence are distinct, but closely related constructs. The last factor, Information-Seeking, is most in line with behavioral indicators of curiosity such as pointing (Lucca and Wilbourn, 2018) or question-asking (Chouinard et al., 2007).

The five factors identified in the Caregiver Activity Subscale encompass a wide spectrum of everyday activities and behaviors that parents integrate into their daily routines. These factors reflect parental activities shown to predict curiosity in older children (e.g., child-led play; Medina and Sobel, 2020) as well as environmental stimulation (i.e., exposure to awe-inducing experiences) that has yet to be studied as a parenting practice, but has an established link with curiosity (Anderson et al., 2020; Paulson et al., 2021).

The CFA results supported our findings from the EFA: both the four-factor structure of the Child Curiosity Subscale and the five-factor structure of the Caregiver Activity Subscale had adequate model fit and plausible substantive interpretations. Since the same data were used for both EFA and CFA, future research should replicate these findings and examine the generalizability of the factor structures of the two subscales in diverse settings using larger samples.

## 4. Study 2B

### 4.1. Introduction

The findings from Study 2A raise the possibility that individual differences in infants’ looking preferences for impossible events might be better understood by examining the *ways* individuals experience and promote curiosity, rather than the sheer degree—certain dimensions of the Child Curiosity Subscale and Caregiver Activity Subscale might uniquely relate to infants’ looking preferences for physically impossible events. Using the factor structures identified in Study 2A, in Study 2B, we explore whether infants’ looking preferences for physically impossible events are predicted by specific aspects of (a) infant everyday curiosity and (b) caregiver curiosity-promoting activities. Since these analyses were exploratory, we did not have prespecified predictions about which dimensions of infant curiosity or caregiver curiosity-promoting activities would relate most strongly to infants’ looking preferences for physically impossible events.

### 4.2. Methods

#### 4.2.1. Participants and procedure

Participants in Study 2B are the same infants from Study 1 with complete questionnaire and looking time data (*N* = 42). We used infants’ looking Preference Score (i.e., average looking preferences for impossible events over the matched possible events across three types of violation events) from Study 1 as our primary outcome variable. The factor structures identified in Study 2A were used to create factor scores. Items within each factor were averaged to calculate a composite score for each factor by averaging the scores on each item under the same factor, leading to four-factor scores in the Child Curiosity Subscale and five-factor scores in the Caregiver Activity Subscale.

### 4.3. Results

We conducted two separate regression models to explore which specific aspects of (1) infant curiosity and (2) caregiver curiosity-promoting activities predicted infants’ looking preferences for physically impossible events.

#### 4.3.1. Child curiosity subscale model

**Figure 5:**
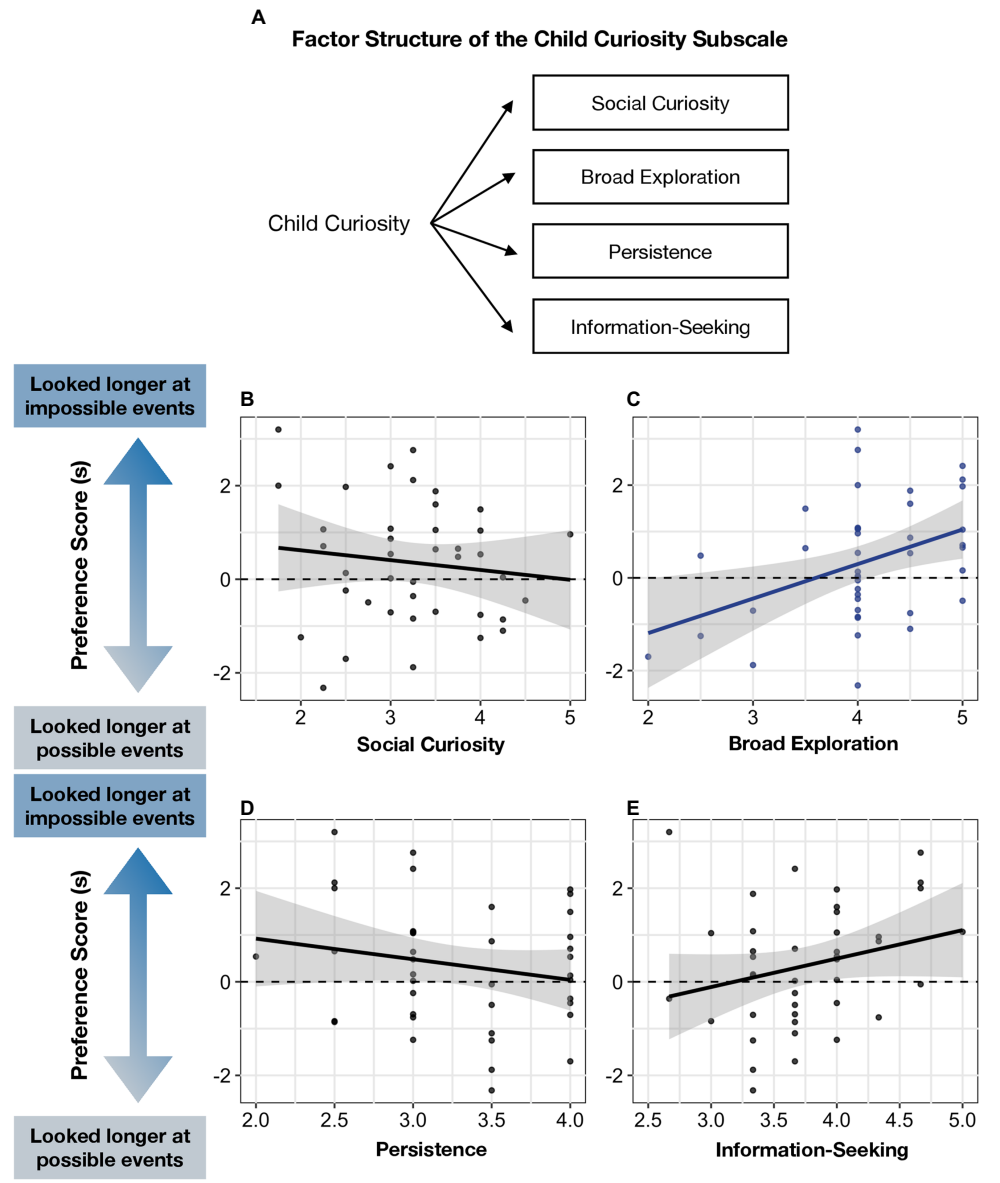
**(A)** Factor structure of Child Curiosity Subscale and **(B–E)** relation between four factors and infants’ Preference Score.

We conducted a multiple regression model with each of the four factors of the Child Curiosity Subscale as predictors (i.e., Social Curiosity, Broad Exploration, Persistence, and Information-Seeking) and infants’ looking preferences as the outcome variable. Infants’ looking preferences were positively predicted by Broad Exploration (*B* = 0.73, *p* = 0.011), but not by the other three factors (all *p*’s > 0.1; see Figure 5 and Table 5).

To explore whether Broad Exploration predicted infants’ looking preferences even after controlling for other factors, we conducted a follow-up regression including infants’ Broad Exploration score, vocabulary size, and temperament as predictors. Infants’ Preference Score was predicted by their Broad Exploration (*B* = 0.80, *p* = 0.006), but not by infants’ vocabulary size or temperament (all *p’s* > 0.1; see Table 6).

**Table 6:** Follow-up multiple regression analysis summary of Child Curiosity Subscale model.

Predictors	*B*	*β*	*t*	*p*	95% CI for *B*
Broad Exploration	0.80**	0.44	2.93	0.006	[0.25, 1.36]
Infant Vocabulary (MCDI)	−0.002	−0.03	−0.16	0.871	[−0.02, 0.02]
Infant Effortful Control (ECBQ)	0.37	0.19	1.18	0.247	[−0.26, 1.00]

**p ≤ 0.01.

#### 4.3.2. Caregiver activity subscale model

**Figure 6:**
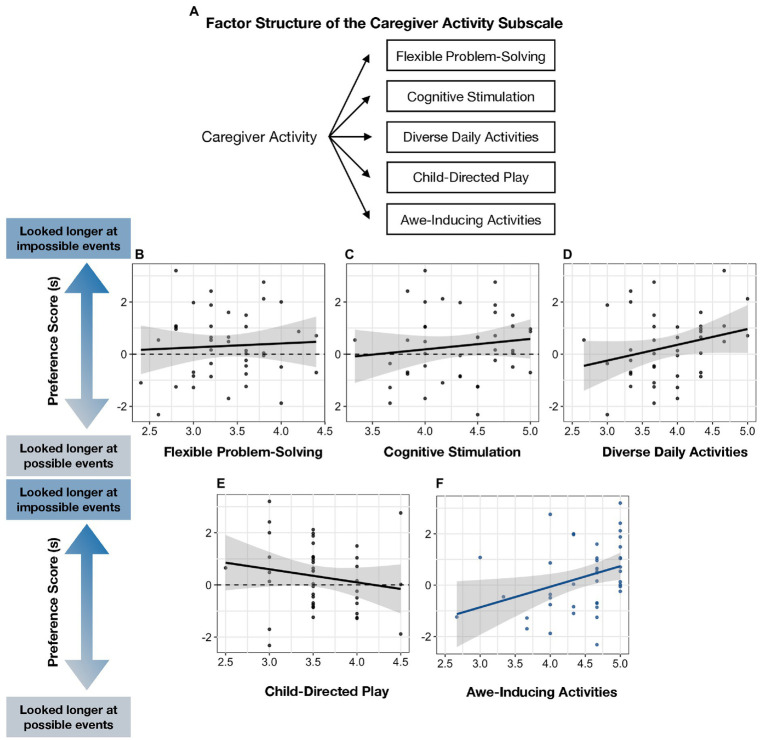
**(A)** Factor structure of Caregiver Activity Subscale and **(B–F)** relation between five factors and infants’ Preference Score.

To explore the unique relations between caregiver curiosity-promoting behaviors and infants’ Preference Score, we conducted a multiple regression model with infants’ five-factor scores on the Caregiver Activity Subscale (i.e., Flexible Problem-Solving, Cognitive Stimulation, Diverse Daily Activities, Child-Directed Play, and Awe-Inducing Activities) as predictors, and infants’ Preference Score as the outcome variable. Awe-Inducing Activities (i.e., taking children to a museum, live performance, and nature walk) were the only caregiver activity score that was positively predicted by infants’ looking preferences for physically impossible events (*B* = 0.77, *p* = 0.034; *p*’s > 0.1 for all other activity scores; see Figure 6 and Table 7).

**Table 7:** Multiple regression analysis summary of Caregiver Activity Subscale model.

Predictors	*B*	*β*	*t*	*p*	95% CI for *B*
Flexible Problem-Solving	0.19	0.07	0.44	0.666	[−0.71, 1.10]
Cognitive Stimulation	0.26	0.09	0.53	0.603	[−0.74, 1.25]
Diverse Daily Activities	0.14	0.06	0.38	0.71	[−0.60, 0.88]
Child-Directed Play	−0.47	−0.16	−1.03	0.311	[−1.40, 0.46]
Awe-Inducing Activities	0.77*	0.34	2.21	0.034	[0.06, 1.47]

*p ≤ 0.05.

To probe whether Awe-Inducing Activities predicted infants’ looking preferences even after controlling for other caregiver factors, we conducted a follow-up regression including caregivers´ Awe-Inducing Activities score, trait curiosity (Joyous Exploration), and selection of curiosity-themed books and toys as predictors. Caregivers´ Awe-Inducing Activities and selection of a curiosity-themed book positively predicted infants´ looking preferences (*B* = 0.77, *p* = 0.041 and *B* = 0.86, *p* = 0.042, respectively), but not caregivers´ trait curiosity or curiosity-themed toy selection (all *p’s* > 0.1; see Table 8).

**Table 8:** Follow-up multiple regression analysis summary of Caregiver Activity Subscale model.

Predictors	*B*	*β*	*t*	*p*	95% CI for *B*
Awe-Inducing Activities	0.77*	0.34	2.12	0.041	[0.03, 1.50]
Caregiver Joyous Exploration (5DC)	0.14	0.09	0.59	0.561	[−0.35, 0.63]
Book Selection	0.86*	0.32	2.11	0.042	[0.03, 1.70]
Toy Selection	0.1	0.03	0.22	0.827	[−0.83, 1.03]

*p ≤ 0.05.

### 4.4. Discussion

In Study 2B, we linked the results from Study 1 and Study 2A to explore predictors of individual differences in infants’ visual preferences for physically impossible events. Among the four dimensions of the Child Curiosity Subscale, only Broad Exploration (i.e., the tendency to explore many toys in a room rather than one) was significantly associated with infants’ looking preferences. In the Caregiver Activity Subscale, only the Awe-Inducing Activities were related to infants’ looking preferences. We also discovered that caregivers’ selection of curiosity-themed books, but not toys, predicted infants’ looking preferences for physically impossible events. We discuss possible explanations for the links between infants’ looking preferences and Broad Exploration, Awe-Inducing Activities, and caregivers’ book selection in the overall discussion.

## 5. Overall discussion

Across two studies, we explored predictors of individual differences in infants’ looking preferences for physically impossible events. We also provided new insights into the nature of early curiosity using a newly developed multidimensional scale, the Early Multidimensional Curiosity Scale (EMCS), designed to measure early curiosity and caregiver curiosity-promoting behaviors. In Study 1, using a violation-of-expectation paradigm and parent surveys, we showed that infants’ looking preferences for physically impossible events were predicted by infants’ everyday curious behaviors and related experiences (i.e., their total score on the EMCS), but not by infants’ temperament, vocabulary, or caregiver trait curiosity. In Study 2A, we analyzed the underlying structure of the EMCS, revealing that both early curiosity and caregivers’ curiosity-promoting activities are multidimensional constructs comprised distinct underlying factors. In Study 2B, we integrated the findings from Study 1 and Study 2A to explore whether certain dimensions of curiosity and caregiver curiosity-promoting behaviors were uniquely related to infants’ looking preferences. We found that children’s Broad Exploration and caregiver’s Awe-Inducing Activities were significant predictors of infants’ looking preferences for physically impossible events.

### 5.1. The multidimensionality of early curiosity and caregiver curiosity-promoting activities

The current study provides a useful new tool, the EMCS, that captures the multifaceted characteristics of early curiosity and related experiences. Using this tool, we show that early curiosity and caregiver curiosity-promoting behaviors consist of distinct subfactors. An exploratory factor analysis revealed that children’s curiosity is comprised four underlying factors, including Social Curiosity, Broad Exploration, Persistence, and Information-Seeking. As described earlier, these factors have meaningful overlap with subfactors of curiosity identified in older children and adults. These findings suggest that curiosity is expressed in multiple ways even from early in life, rather than gradually differentiated across development, either due to cognitive maturation or relevant experiences. Though other important components of adult curiosity, such as thrill seeking and risk taking, were beyond the scope of our survey, future research should explore whether these are also early emerging components of curiosity, or whether these aspects of curiosity only emerge until later in development. Relatedly, caregiver curiosity-promoting activities consisted of five unique factors, including Flexible Problem-Solving, Cognitive Stimulation, Diverse Daily Activities, Child-Directed Play, and Awe-Inducing Activities. These factors capture a range of everyday activities, informal teaching practices, and parenting styles that may play an important role in the development of early curiosity.

Though the dimensionality of early curiosity and caregiver curiosity-promoting activities identified here relates to previous findings (Litman and Mussel, 2013; Colantonio and Bonawitz, 2018; Medina and Sobel, 2020; Evans et al., 2021), the generalizability of these factor structures should be examined in more diverse settings. For example, though the two-factor structure of epistemic curiosity (i.e., interest-focused (I-Type) and deprivation-focused (D-Type); Litman and Spielberger, 2003), Karandikar et al. (2021) identified in adults from the United States showed a good fit for adults from India, the ways in which these two types of epistemic curiosity were related to other relevant constructs were different across the two samples. I-Type curiosity was positively correlated with agreeableness and extraversion in the US sample, but not in the Indian sample. Moreover, young children in different cultures have different information-seeking styles (Gauvain et al., 2013), suggesting that cultural variability in curiosity might be captured early in life. Relatedly, though research has yet to directly examine cultural differences in caregiver curiosity-promoting behaviors, caregivers differ around the world in parenting practices and socialization goals, which may, in turn, influence curiosity-promoting behaviors, such as activities tailored to the development of exploration (Little et al., 2016), self-esteem (Miller et al., 2002), and autonomy (Keller et al., 2007). In sum, future research is needed outside the context of the United States to investigate the generalizability or distinctiveness of the factor structures identified here.

### 5.2. Infants’ everyday broad exploration and their looking preferences for physically impossible events

Though previous research has linked infants’ interest in physically impossible events to curiosity later in childhood (Perez and Feigenson, 2021), questions remained about whether infants’ looking time to unexpected events reflected their *selective* interest in physically impossible events during infancy. Here, we more closely examined the relation between infants’ looking preferences for physically impossible events and everyday curiosity in infancy. We ruled out potential confounding factors by measuring the relation between infants’ looking preferences for physically impossible events and their everyday curiosity *concurrently* and with other psychological factors that were not controlled for in previous research (Perez and Feigenson, 2021). We focused on infants’ temperament (effortful control) in particular, because it is an indicator of infants’ attentional control (i.e., their ability to focus attention on tasks; Rothbart et al., 2000) and may play an important role in guiding their visual attention to physically impossible events.

Another key control variable included in the current study was infants’ attention to possible events. We generated a Preference Score by calculating the difference between infants’ looking time to impossible versus possible events, which allowed us to rigorously capture infants’ selective interests in impossible events over possible events. Though we used an average Preference Score of three physical events (i.e., Relocation, Occlusion, and Solidity) for the main regression analyses, in the breakdown analysis of infants’ looking preferences, we detected that infants’ looking Preference Score in the Solidity event was significantly higher than Relocation and Occlusion events. The visual simplicity of the Solidity event (e.g., unlike the Relocation and Occlusion events, the features of the familiarization and test trials were nearly identical in the Solidity event) or gradual learning in study cues (e.g., structure of trials/events) because of the fixed order of events may have also contributed to this phenomenon (since Solidity was always presented last). Future research is needed to disentangle whether and how specific types of physical violations are associated with infants’ curiosity. Among the four factors identified in the child subscale of the EMCS, only Broad Exploration was associated with infants’ looking preferences for physically impossible events. This finding suggests that broad exploration may be a powerful catalyst of curiosity during infancy because it exposes them to new information (e.g., physical principles that govern how objects and toys work) in a short period of time. Such heightened knowledge about and experience with their environment may, in turn, drive infants’ interest in learning about the world. Previous research has similarly revealed a selective association between infants’ looking preferences and subfactors of curiosity. Perez and Feigenson (2021) found that infants’ looking preference for physically impossible events at 17 months related to deprivation-focused (D-Type) curiosity (i.e., curiosity motivated by reduction of uncomfortable feelings associated with experiencing a gap in knowledge) measured at 36 months. Together, these findings suggest that infants’ looking preferences have specific relations with distinct forms of curiosity. Yet, more research is needed to disentangle the drivers of different types of curiosity during infancy and how they differentially relate to infants’ looking preferences for impossible events across different time points in development.

Since our violation-of-expectation tasks assessed infants’ interest in the physical world, it is not surprising that infants’ everyday broad exploration of toys, but not aspects of curiosity less related to physical exploration, was positively associated with their looking preferences for physically impossible events. It is possible that other aspects of everyday curiosity unrelated to the physical world might correlate to infants’ looking preferences in other contexts. For instance, infants’ everyday social curiosity might be uniquely related to infants’ looking preferences for events in which social norms are violated (e.g., violations of fairness norms; Ziv and Sommerville, 2017; violations of relationship norms; Liberman et al., 2014).

### 5.3. The relation between parenting practices and infant curiosity

The current study also demonstrates that, just like curiosity itself, the ways caregivers set out to foster curiosity in young children are diverse and multifaceted. Among the five dimensions of curiosity-promoting activities we identified in the EMCS, only Awe-Inducing Activities (i.e., taking children on nature walks, to live performances, or to museums) were associated with infants’ preferences for physically impossible events. Critically, however, caregiver trait curiosity did not relate to infants’ looking preferences for physically impossible events – a finding that underscores the importance of parent–child interactions in the development of early curiosity.

Though past work has identified a relation between awe and curiosity later in development (Colantonio and Bonawitz, 2018; McPhetres, 2019; Anderson et al., 2020), our findings provide the first evidence that awe-inducing experiences provided by parents relate to curiosity in infancy. What mechanism might support a link between caregiver awe-inducing activities, and infants’ looking preferences for physically impossible events? It is possible that infants who are more curious about the world around them experience more enjoyment during awe-inducing activities, and therefore have caregivers who tend to engage these activities more frequently. Alternatively, it may be that the experience of awe triggers curiosity. The experience of awe is often characterized by a sensation of perceptual vastness. Encountering vastness, either in the environment (e.g., visiting the Grand Canyon) or in others (e.g., meeting someone you admire), leads individuals to perceive themselves as small because it raises awareness that the world is full of new information. It has been proposed that uncertainty elicited by this contrast provokes a need for accommodation, ultimately triggering a drive to extend and/or change existing knowledge structures (Keltner and Haidt, 2003). In support of this, in one study, preschool children who were shown awe-inducing videos reported a smaller perceived self-size, and subsequently heightened exploration relative to children shown happy- or calm-inducing videos (Colantonio and Bonawitz, 2018). Thus, it is possible that infants whose caregivers engage in awe-inducing activities tend to be more sensitive to gaps in their knowledge, which may, in turn, lead them to show greater looking preferences for physically impossible events. The current study is unable to disentangle these hypotheses, and future research is needed to elucidate the nature of the relation between awe and curiosity during infancy.

The current study also found that caregivers’ selection of curiosity-themed books, but not toys, was significantly related to infants’ looking preferences for physically impossible events. Prior research has shown that caregivers tend to engage in more pedagogical parenting practices during book, compared to toy, interactions (Salo et al., 2016). For example, during book reading interactions, compared to joint toy play, both caregivers and their 12-month-old infants engage in more sophisticated verbal and non-verbal communicative interactions as measured by increased complexity in language use, vocabulary, and syntax (Yont et al., 2003). Indeed, caregivers of infants in our study selected curiosity-themed books at higher rates (66% of caregivers selected curiosity-themed books, 34% of caregivers selected books with non-curiosity-themed books) than curiosity-themed toys (only 26% of caregivers selected curiosity-themed toys, 74% of caregivers selected non-curiosity-themed toys). Together, these findings suggest that caregivers may view books as a unique opportunity for socializing children about curiosity.

## 6. Limitations and future directions

One open question concerns the degree to which the parental reports described here relate to infants’ actual everyday curiosity and caregivers’ real-world engagement in curiosity-promoting behaviors. Multi-method research is needed to test whether factors of early curiosity identified in parental reports can be directly detected through infants’ behaviors and preferences, and whether parents’ curiosity-promoting behaviors in real life are indeed correlated with their responses on the EMCS.

Because this study was conducted over Zoom during the COVID-19 pandemic, our sample had limited socioeconomic diversity (88% of parents across both studies had a college degree or higher). Socioeconomic status may shape the type of activities parents provide for their children. For example, awe-inducing activities may require more financial flexibility (e.g., buying a ticket for a live performance, traveling to or living in proximity to nature) than other types of curiosity-related activities (e.g., asking children open-ended questions). Future research should examine whether and how parental activities vary depending on family demographics, and whether the effects of different dimensions of caregiver curiosity-promoting activities on child’s curiosity also vary depending on various family factors.

## 7. Conclusion

Like “talent” and “brilliance,” “curiosity” is often portrayed as a natural gift that some of us have more than others. And just like “talent” and “brilliance,” “curiosity” is also thought to be critical for making significant intellectual contributions to human society. Understanding the nature of curiosity may not only influence how we approach childhood learning and education, but also how we perceive it in ourselves—both in terms of whether we feel we “deserve” to be in a profession that requires high levels of curiosity (in a way that is similar to how we perceive “talent” and “brilliance” influences us; Leslie et al., 2015) and how stereotypes are developed about professions requiring high levels of curiosity that further impact those perceptions. Fortunately, curious cognitive scientists are building an emerging body of work to understand what makes us curious and why. Our work contributes to this broader mission by identifying potential sources of individual differences in curiosity, as well as new tools to measure those differences, right as they begin to emerge in the first few years of life.

## Data availability statement

The datasets presented in this study can be found in online repositories. The names of the repository/repositories and accession number(s) can be found at: https://osf.io/dnzty/?view_only=0f6c14b5eb8f4a17a65f20b3a4b1289b.

## Ethics statement

The studies involving human participants were reviewed and approved by Ethics Committee at Arizona State University. Written informed consent to participate in this study was provided by the participants’ legal guardian/next of kin.

## Author contributions

NL, VL, and KL developed the study concept and contributed to the study design. Testing and data collection were performed by VL. Data analysis, interpretation, and drafting of the manuscript were performed by NL, VL, JW, HHŞ, and KL. All authors contributed to the article and approved the submitted version.

## Funding

This work was supported by funding from the Department of Psychology at Arizona State University.

## Conflict of interest

The authors declare that the research was conducted in the absence of any commercial or financial relationships that could be construed as a potential conflict of interest.

## Publisher’s note

All claims expressed in this article are solely those of the authors and do not necessarily represent those of their affiliated organizations, or those of the publisher, the editors and the reviewers. Any product that may be evaluated in this article, or claim that may be made by its manufacturer, is not guaranteed or endorsed by the publisher.

## Footnotes

^1^ ChildrenHelpingScience.com

^2^ www.datavyu.org
