# Supplementary material for: Exploring individual differences in infants’ looking preferences for impossible events: The Early Multidimensional Curiosity Scale
Source: Front Psychol. 2023 Feb 2;13:1015649. doi: 10.3389/fpsyg.2022.1015649 (PMC9931910; doi:10.3389/fpsyg.2022.1015649)
Supplement: Supplementary file 2 [file Data_Sheet_1.pdf]

Supplementary Material

1. Supplementary Methods

The data used in Study 1 were pulled from a larger study investigating the development of active learning. After participants watched the warm-up video, they watched a series of four other videos prior to the violation-of-expectation task videos: a woman blowing bubbles (1 min); dancing light video (1 min); visual patterns with varying levels of complexity (2 min); and an object labeling video with human actors (3 min).

2. Supplementary Results

2.1 Looking Time Trial-Level Analyses

	Relocation M_{sec} (SD)	Occlusion M_{sec} (SD)	Solidity M_{sec} (SD)
Possible event	7.57 (2.63) (n = 40)	7.96 (1.76) (n = 44)	6.80 (2.35) (n = 43)
Impossible event	7.63 (2.66) (n = 40)	7.65 (2.07) (n = 43)	7.77 (2.69) (n = 42)

Possible test events. Infants' looking time in the Occlusion test trial was highly correlated with their looking time in the Solidity test trial ($r = 0.32$, $p < .05$); infants' looking time in the Occlusion test trial was highly correlated with their looking time in the Relocation test trial ($r = 0.59$, $p < .001$); infants' looking time in the Solidity test trial was positively, but not significantly, correlated with their looking time in the Relocation test trial ($r = 0.27$, $p = .091$).

Impossible test events. Infants' looking time to the impossible test events was highly correlated across all test trials (r = 0.34 to 0.47, all p's < .05).

Though infants' looking time was highly correlated across trials (i.e., across three impossible events and possible events), their Preference Score was not positively correlated across trials (r = -0.36 to -0.05, all p's > .05).

2.2 Order effects

One-way repeated measures of ANOVA were conducted to test for order effects – whether infants' Preference Scores decreased across three events. The results showed that the order of three events had a statistically significant effect on infants' Preference scores ($F(2, 48)$ = , $p = 0.049$). Post hoc tests using Tukey's HSD revealed that infants' Preference Score was significantly higher in the third event (i.e., Solidity) ($M_{sec} = 1.34$) than the first (Relocation, $M_{sec} = 0.06$) and the second event (Occlusion, $M_{sec} = -0.27$) (all p's < .05). There was no significant difference between the Relocation and Occlusion trial (p > .05). Though the order effect was significant, it did not affect our data in a way it is usually concerned in infant research adopting a looking time measure (i.e., the effect got stronger, rather than weaker, across trials).

Appendix

Appendix A. The Early Multidimensional Curiosity Scale (EMCS)

Items marked with an **[R]** are reverse coded.
Items marked with an asterisk are modified in the Child Curiosity Subscale: Child version (for use with children 2 years and older).
Items marked with a double asterisk are only included in the Child Curiosity Subscale: Child version (for use with children 2 years and older).
The Caregiver Activity Subscale is used in both age groups: infants (2 years and younger) and children (2 years and older).

Caregiver Activity Subscale

This set of questions asks about how you interact and play with your child. Please answer each question to the best of your ability.

1. When your child is learning something new (for example, how to play with a new toy), how often do you…
   a. Encourage them to figure out how it works on their own?
      i. Never
      ii. Almost Never
      iii. Sometimes
      iv. Almost Always
      v. Always
   b. Provide them with everything they need to learn, by telling or showing them what to do? **[R]**
      i. Never
      ii. Almost Never
      iii. Sometimes
      iv. Almost Always
      v. Always
2. Think of some of your favorite hobbies. When you do these hobbies, how often do you show your child what you are doing or take them with you?
   a. Never
   b. Almost Never
   c. Sometimes
   d. Almost Always
   e. Always
3. Open-ended questions are questions that require more than a single word answer and encourage children to think deeply. How often do you ask your child open-ended questions?
   a. Never
   b. Almost Never
   c. Sometimes
   d. Almost Always

    e. Always
4. During play time, examples of child leading behaviors include: independently picking toys to play with, making their own decisions, physically interacting with toys without the caregiver starting the game. How often does your child lead play time?
    a. Never
    b. Almost Never
    c. Sometimes
    d. Almost Always
    e. Always
5. During play time, examples of behaviors parent leading behaviors include: providing your child with instructions, making decisions for your child, showing your child what to do/how to do something. How often do <u>you</u> lead play time? **[R]**
    a. Never
    b. Almost Never
    c. Sometimes
    d. Almost Always
    e. Always
6. How interested would you be in taking your child...[1]
    a. To a museum (such as a science museum, art museum, history museum)?
        i. Extremely Uninterested
        ii. Somewhat Uninterested
        iii. Neutral
        iv. Somewhat Interested
        v. Extremely Interested
    b. On a nature walk or hike with you?
        i. Extremely Uninterested
        ii. Somewhat Uninterested
        iii. Neutral
        iv. Somewhat Interested
        v. Extremely Interested
    c. To a live performance (such as a concert, magic show, theater)?
        i. Extremely Uninterested
        ii. Somewhat Uninterested
        iii. Neutral
        iv. Somewhat Interested
        v. Extremely Interested

---

[1] This survey was administered at the start of the COVID-19 pandemic in 2020 when indoor and outdoor activities were broadly restricted. Therefore, we asked caregivers about their interest in engaging in activities included in 6a-6c, rather than asked their actual experience. Future studies may consider reframing these questions and asking caregivers' frequency in engaging in those activities, such as "How often do you take your child to a museum?".

Supplementary Material

How frequently do you do the following behaviors or activities?

Very Frequently, Frequently, Sometimes, Almost never, or Never

7. Introduce your child to:
    a. Unfamiliar objects (like whisks) or new toys?
    b. New people (such as friends, extended family members, strangers)?
    c. New places (such as hair salons/barber shops, laundromats, your workplace, movie theater)?
    d. New cultures or languages (through videos, TV shows, books)?
8. Show your child different types of activities (for example, art, sports, cooking, music)?
9. When you and your child are outside, talk about your surroundings (such as plants, animals, rocks, mountains, clouds)?
10. When playing with your child, use objects or toys for something outside of their intended purpose (for example, playing a pot like a drum or wearing it like a hat)?
11. When your child expresses interest in getting new information (through asking questions, gesturing to an object, or looking at you), provide detailed, in-depth answers?
12. When your child is trying to play with a new toy, and they grow frustrated or discouraged with an activity, encourage them to move on to another toy (as opposed to stick with the original activity)? **[R]**

How much do you agree or disagree with the following statements?

Strongly Disagree, Disagree, Neither agree nor disagree, Agree, or Strongly agree

13. I would rather take my child to familiar places, than new places we haven't been before. **[R]**
14. When solving a new problem, I encourage my child to use the strategy I would choose to use, rather than allowing them to try alternative strategies I would not use. **[R]**
15. I encourage my child to use their imagination.
16. I discourage my child from exploring their surroundings. **[R]**
17. I want to encourage my child's interests, even if I don't share those interests.
18. I encourage my child to participate in creative activities (such as building blocks, pretend-play, or making music).
19. When something happens that violates my child's expectations about the world, I draw their attention to it.
20. I allow my child to try new ways of playing with things, even when it may not be what those items were designed for.
21. I encourage my child to try doing new things they have never done before.
22. I encourage my child to think about how others are feeling.
23. I would rather read a familiar book to my child (one we've read before) than one that we have not read before. **[R]**
24. I encourage my child to try new foods.
25. I encourage my child to look at problems from multiple perspectives.
26. I try to draw my child's attention to things they don't know.
27. I encourage my child to embrace uncertainty.

Child Curiosity Subscale: Infant Version
(for use with children 2 years and younger)

This set of questions asks about how your child behaves, learns, and plays. Please answer each question to the best of your ability.

1. How often does your child point, gesture, or talk about things that are new to them?*
   a. Never
   b. Almost Never
   c. Sometimes
   d. Almost Always
   e. Always
2. When presented with unfamiliar objects (things they have never seen before), how often does your child point, gesture, or talk about things that are new to them?*
   a. Never
   b. Almost Never
   c. Sometimes
   d. Almost Always
   e. Always
3. How often does your child independently discover creative uses for objects outside of their intended purpose (example: using a book to build a tower)?
   a. Never
   b. Almost Never
   c. Sometimes
   d. Somewhat frequently
   e. Frequently
4. When something contradicts what your child knows about the world, how often does your child further explore, as if to figure out what happened?
   a. Never
   b. Almost Never
   c. Sometimes
   d. Almost Always
   e. Always
5. When your child has difficulty completing a task, to what extent…
   a. ...are they highly motivated to keep trying?
      i. Never
      ii. Almost Never
      iii. Sometimes
      iv. Almost Always
      v. Always
   b. ...do they give up immediately and shift their attention to another task? **[R]**
      i. Never

ii. Almost Never
iii. Sometimes
iv. Almost Always
v. Always

c. ...do they get upset if interrupted? **[R]**

i. Never
ii. Almost Never
iii. Sometimes
iv. Almost Always
v. Always

6. When playing with a new toy, how often does your child communicate with you as if requesting help or further instruction on what to do with the new toy?
   a. Never
   b. Almost Never
   c. Sometimes
   d. Almost Always
   e. Always
7. When visiting a new environment (such as a friend's home, playground, or park), how often...
   a. ...does your child quickly gravitate toward other children?
      i. Never
      ii. Almost Never
      iii. Sometimes
      iv. Almost Always
      v. Always
   b. ...does your child immediately go to where there are many people (versus more isolated areas)?
      i. Never
      ii. Almost Never
      iii. Sometimes
      iv. Almost Always
      v. Always
8. Imagine that you present your child with a brand new toy and one they are already familiar with. How likely is your child to…
   a. Prefer playing with the familiar toy over the new toy? **[R]**
      i. Extremely Unlikely
      ii. Unlikely
      iii. Neutral
      iv. Likely
      v. Extremely Likely
9. Imagine your child walks into a new playroom filled with different kinds of toys. How likely is your child to…
   a. Spend the whole time playing with one specific toy? **[R]**

i. Extremely Unlikely
ii. Unlikely
iii. Neutral
iv. Likely
v. Extremely Likely

b. Explore the majority of toys in the room?
   i. Extremely Unlikely
   ii. Unlikely
   iii. Neutral
   iv. Likely
   v. Extremely Likely

10. To what extent does your child like playing with toys or playing games that involve elements of surprise (such as a jack-in-the-box, peek-a-boo)?*
    a. Extremely Dislikes
    b. Somewhat Dislikes
    c. Neither likes nor Dislikes
    d. Somewhat Likes
    e. Extremely Likes
11. To what extent does your child enjoy interacting with…
    a. new adults?
       i. Extremely Dislikes
       ii. Somewhat Dislikes
       iii. Neither likes nor Dislikes
       iv. Somewhat Likes
       v. Extremely Likes
    b. new children?
       i. Extremely Dislikes
       ii. Somewhat Dislikes
       iii. Neither likes nor Dislikes
       iv. Somewhat Likes
       v. Extremely Likes
12. Babies experience many new events or “firsts” in the first few years of life. Select how interested your baby was during the following “firsts”:
    Please select “NA” if your child has never experienced the scenario, or you don’t recall how they responded.
    a. They see a familiar person change their physical appearance (for example by cutting their hair, shaving a beard, or wearing a costume)*:
       i. Very uninterested
       ii. Somewhat uninterested
       iii. Indifferent
       iv. Somewhat interested
       v. Very interested

vi. NA

b. They see something break or spill*:
   i. Very uninterested
   ii. Somewhat uninterested
   iii. Indifferent
   iv. Somewhat interested
   v. Very interested
   vi. NA

c. They taste something new*:
   i. Very uninterested
   ii. Somewhat uninterested
   iii. Indifferent
   iv. Somewhat interested
   v. Very interested
   vi. NA

d. They touch something new (for example, feeling a new texture)*:
   i. Very uninterested
   ii. Somewhat uninterested
   iii. Indifferent
   iv. Somewhat interested
   v. Very interested
   vi. NA

e. They interact with a new electronic gadget (like an automatic vacuum, Alexa, or Siri)*:
   i. Very uninterested
   ii. Somewhat uninterested
   iii. Indifferent
   iv. Somewhat interested
   v. Very interested
   vi. NA

f. They crawl or walk on a new surface*:
   i. Very uninterested
   ii. Somewhat uninterested
   iii. Indifferent
   iv. Somewhat interested
   v. Very interested
   vi. NA

Child Curiosity Subscale: Child Version
(for use with children 2 years and older)

1. How often does your child ask questions or talk about things that are new to them?*
   a. Never
   b. Almost Never
   c. Sometimes
   d. Almost Always
   e. Always
2. When presented with unfamiliar objects (things they have never seen before), how often does your child approach them on their own?*
   a. Never
   b. Almost Never
   c. Sometimes
   d. Almost Always
   e. Always
3. Imagine that you present your child with a brand new toy and one they are already familiar with. How likely is your child to…
   a. Prefer playing with the familiar toy over the new toy? **[R]**
      i. Extremely Unlikely
      ii. Unlikely
      iii. Neutral
      iv. Likely
      v. Extremely Likely
4. Imagine your child walks into a new playroom filled with different kinds of toys. How likely is your child to…
   a. Spend the whole time playing with one specific toy? **[R]**
      i. Extremely Unlikely
      ii. Unlikely
      iii. Neutral
      iv. Likely
      v. Extremely Likely
   b. Explore the majority of the toys in the room?
      i. Extremely Unlikely
      ii. Unlikely
      iii. Neutral
      iv. Likely
      v. Extremely Likely
5. How often does your child independently discover creative uses for objects outside of their intended purpose (example: using a book to build a tower)?
   a. Never
   b. Almost Never

   c. Sometimes
   d. Somewhat frequently
   e. Frequently
6. How often does your child (on their own) engage in pretend play (example: playing house, playing doctor, etc.)?**
   a. Never
   b. Almost Never
   c. Sometimes
   d. Somewhat frequently
   e. Frequently
7. When something contradicts what your child knows about the world, how often does your child further explore, as if to figure out what happened?
   a. Never
   b. Almost Never
   c. Sometimes
   d. Almost Always
   e. Always
8. When your child has difficulty completing a task, to what extent…
   a. ...are they highly motivated/happy to keep trying?
      i. Never
      ii. Almost Never
      iii. Sometimes
      iv. Almost Always
      v. Always
   b. ...do they give up immediately and shift their attention to another task? **[R]**
      i. Never
      ii. Almost Never
      iii. Sometimes
      iv. Almost Always
      v. Always
   c. ...do they get upset if bothered or interrupted? **[R]**
      i. Never
      ii. Almost Never
      iii. Sometimes
      iv. Almost Always
      v. Always
9. To what extent does your child like playing with toys or playing games that involve elements of surprise (for example, toys that have items pop out or mystery games)?*
   a. Extremely Dislikes
   b. Somewhat Dislikes
   c. Neither likes nor Dislikes
   d. Somewhat Likes

e. Extremely Likes

10. Think about a recent time your child encountered the following. How interested were they in the experience? (If your child has not experienced the following, or you do not remember their response, select NA).
    a. A natural wonder (Grand Canyon, waterfall, planets through a telescope, oceans, etc)*:
        i. Very uninterested
        ii. Somewhat uninterested
        iii. Indifferent
        iv. Somewhat interested
        v. Very interested
        vi. NA
    b. A magic trick*:
        i. Very uninterested
        ii. Somewhat uninterested
        iii. Indifferent
        iv. Somewhat interested
        v. Very interested
        vi. NA
    c. A new science-based toy, experiment, or museum exhibit (for example: kinetic sand, telescopes, grow-in-water toys)*:
        i. Very uninterested
        ii. Somewhat uninterested
        iii. Indifferent
        iv. Somewhat interested
        v. Very interested
        vi. NA
    d. Mystery-related books, games, or riddles*:
        i. Very uninterested
        ii. Somewhat uninterested
        iii. Indifferent
        iv. Somewhat interested
        v. Very interested
        vi. NA
    e. Extreme weather (snow, thunder, lightning, fail, earthquake, etc)* :
        i. Very uninterested
        ii. Somewhat uninterested
        iii. Indifferent
        iv. Somewhat interested
        v. Very interested
        vi. NA
    f. A plant, insect, or animal they have never seen before*:

i. Very uninterested
ii. Somewhat uninterested
iii. Indifferent
iv. Somewhat interested
v. Very interested
vi. NA

11. How often does your child get bored/uninterested in a toy/activity?**
    a. Never
    b. Almost Never
    c. Sometimes
    d. Almost Always
    e. Always
12. To what extent does your child enjoy interacting with…
    a. new adults?
        i. Extremely Dislikes
        ii. Somewhat Dislikes
        iii. Neither likes nor Dislikes
        iv. Somewhat Likes
        v. Extremely Likes
    b. new children?
        i. Extremely Dislikes
        ii. Somewhat Dislikes
        iii. Neither likes nor Dislikes
        iv. Somewhat Likes
        v. Extremely Likes
13. When visiting a new environment (such as a friend's home, playground, or park), how often...
    a. ...does your child quickly gravitate toward other children?
        i. Never
        ii. Almost Never
        iii. Sometimes
        iv. Almost Always
        v. Always
    b. ...does your child immediately go to where there are many people (versus more isolated areas)?
        i. Never
        ii. Almost Never
        iii. Sometimes
        iv. Almost Always
        v. Always
    c. …is your child comfortable playing with new people?**
        i. Never
        ii. Almost Never

iii. Sometimes
iv. Almost Always
v. Always

14. When playing with a new toy or game, how often does your child communicate with you as if requesting help or further instruction on what to do with the new toy/game?
    a. Never
    b. Almost Never
    c. Sometimes
    d. Almost Always
    e. Always

Supplementary Material

Appendix B. Caregivers' Preferences for Curiosity-Themed Books and Toys

Curiosity-Themed Book Choice Measure. Parents were given the following prompt and asked to select on the three options. "I Wonder" was considered a "curiosity" selection, the other two were not.

We are also interested in learning what types of books parents are most interested in for their children. If you were to select a new book for your child to have, which one would you pick?

- ***Being Grateful:*** Following Jordan and friends through the course of a day, this book affirms the significance of helping others and showing gratitude. Jordan discovers how to share toys in the classroom, help someone find their missing dog, and be grateful for others' acts of kindness! This book shows children the true meaning of compassion and cheering others on.

- ***I Wonder***: One day, Alex takes a walk and encounters a range of mysteries: from gravity, to life cycles, to the vastness of the universe. "How does the moon stay so close? Where does gravity come from?" Alex discovers that there are some things even adults don't know—mysteries for everyone to wonder about together! This book celebrates curiosity in children as the foundation for learning.

- ***Counting Celebration:*** It's another busy day for Taylor -- Taylor and friends are deciding to plan a special birthday party for their next door neighbor. They need help counting the items they need for their guests—how many balloons should there be?! Learn how to count from 1 to 10 in this fun and enjoyable book! This book will help develop children's early mathematical learning.

Curiosity-Themed Toy Choice Measure. Parents were given the following prompt and asked to select on the three options. "Exploration Station" was considered a "curiosity" selection, the other two were not.

We are in the process of buying new toys for our lab. Before you go, we would appreciate your feedback on what toys babies and toddlers would be most interested in! If you were to select a new toy for your child to have, which one would pick?

- ***Music Box:*** Press a button on each side of the box to hear your child's favorite instruments such as the piano, guitar, and drums! Move and groove with your child to 6 popular tunes! What's your favorite instrument on the Music Box?

- ***Hungry Mr. Monkey:*** Mr. Monkey is always hungry for some bananas. Press the big blue button to feed Mr. Monkey! The more bananas you feed Mr. Monkey, the happier he gets! How many bananas can you feed Mr. Monkey?

- ***Exploration Station***: Let’s explore and make new discoveries! Rotate the station to reveal new sea creatures, and press the green button to hear their names, where they live, and a fun fact! How many new discoveries can you make?
